# Prognostic value of circulating proteins in patients undergoing surgery for pancreatic cancer

**DOI:** 10.1002/cam4.5240

**Published:** 2022-10-17

**Authors:** Sidsel C. Lindgaard, Zsófia Sztupinszki, Emil Maag, Carsten P. Hansen, Inna M. Chen, Astrid Z. Johansen, Jane P. Hasselby, Stig E. Bojesen, Dorte Nielsen, Julia S. Johansen

**Affiliations:** ^1^ Department of Oncology Copenhagen University Hospital–Herlev and Gentofte Herlev Denmark; ^2^ Danish Cancer Society Research Center Copenhagen Denmark; ^3^ BioXpedia Aarhus Denmark; ^4^ Department of Surgery Copenhagen University Hospital – Rigshospitalet Copenhagen Denmark; ^5^ Department of Pathology Copenhagen University Hospital – Rigshospitalet Copenhagen Denmark; ^6^ Department of Clinical Biochemistry Copenhagen University Hospital ‐ Herlev and Gentofte Herlev Denmark; ^7^ Institute of Clinical Medicine, Faculty of Health and Medical Sciences University of Copenhagen Copenhagen Denmark; ^8^ Department of Medicine Copenhagen University Hospital ‐ Herlev and Gentofte Herlev Denmark

**Keywords:** biomarkers, pancreatic cancer, proteins, surgery

## Abstract

**Background:**

Pancreatic ductal adenocarcinoma (PDAC) is the fourth leading cause of cancer death. Less than 20% of patients are diagnosed with resectable disease. Identifying truly resectable disease is challenging because 20%–40% of the patients subjected to resection are found to have advanced disease during surgery. The aim of our study was to identify panels of circulating proteins that could be used to distinguish patients with unresectable PDAC from patients with resectable PDAC and to identify prognostic signatures for both groups.

**Methods:**

We measured 92 circulating immuno‐oncology‐related proteins using the proximity extension assay from Olink Proteomics in 273 patients eligible for surgery for PDAC. Two bioinformaticians worked independently of one another on the same data. LASSO and Ridge regression were used in the statistical analyses.

**Results:**

One protein index for determining resectability had an AUC value of 0.66. Several indices for prognosis had AUC values between 0.50 and 0.75 and were therefore not better than existing prognostic markers.

**Discussion:**

Our study did not reveal any new high‐performing protein panels that could be used to identify patients with inoperable PDAC before surgery. The panel of 92 proteins investigated has previously been found to be applicable for diagnostic use in patients with PDAC, but it does not seem to warrant further investigation regarding resectability in the subgroup of patients with PDAC referred to surgery.

## Graphical abstract

Determining resectability and prognosis in pancreatic ductal adenocarcinoma (PDAC) can be difficult. We examined the capabilities of 92 circulating proteins for the determination of resectability and prognosis in 273 patients undergoing surgery for PDAC.

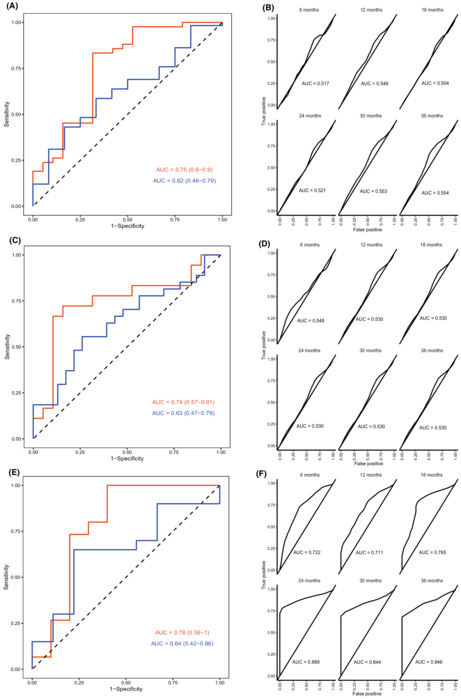

## 1 INTRODUCTION

Pancreatic ductal adenocarcinoma (PDAC) is currently the fourth leading cause of cancer‐related death and is projected to be the second leading cause by 2030.^1^, ^2^ The majority of patients have locally advanced or metastatic disease at diagnosis, and <20% of the patients are eligible for resection.^3^, ^4^ Resection remains the only potentially curative treatment for PDAC, but approximately 80% of resected patients with PDAC relapse within 2 years.^5^, ^6^

Determining resectability in PDAC can be difficult. Computed tomography (CT) scan is the preferred imaging modality for the diagnosis and staging of PDAC.^7^ Yet, even when assessed to be resectable on a CT scan, patients may be found to have unresectable disease during surgery. A Cochrane review from 2016 found that 41.4% of patients subjected to surgery based on CT scans had unresectable disease during surgery.^8^ However, the percentage of patients with accurate upfront staging of resectable PDAC has increased in recent years. Recent data from the Danish Pancreatic Cancer Database reveal that 79% of planned resections for PDAC are completed.^9^ This still leaves around 20% of patients where the determination of resectability was inadequate. Thus, better selection of patients for surgery is needed.

To reduce the risk of recurrence, guidelines recommend 6 months of adjuvant chemotherapy after resection for PDAC.^10^ However, patients must have a good performance status to receive chemotherapy. Many patients never fully recover from surgery due to either surgical complications or occult metastatic disease at the time of resection and rapidly deteriorate post‐surgery.^11^ Between 50% and 65% of patients never receive chemotherapy after resection, and a recent study found that < 10% of the patients complete the full 6 months of recommended adjuvant chemotherapy after resection for PDAC.^11^, ^12^

A biomarker could potentially aid in the identification of patients eligible for surgery. CA19‐9 has been shown to be a predictor of resectability as well as a prognostic biomarker both pre‐operatively and longitudinally after resection.^13^, ^14^, ^15^, ^16^ However, CA19‐9 is not specific for PDAC and can be elevated in other types of cancer (e.g. biliary tract and colorectal cancer) and some benign conditions (e.g. pancreatitis and cholestasis), and approximately 10% of the general population are unable to synthesize CA19‐9.^14^, ^17^

Inflammation is a key player in tumor development and a driver of tumor growth and metastasis in PDAC.^18^, ^19^ A systemic inflammatory response with circulating monocytes, increased neutrophil to lymphocyte ratios, and increased cytokines and CRP levels can be detected in PDAC with and without metastases.^19^ Within the PDAC tumor micro‐environment, there is a complex mixture of extracellular matrix, suppressive immune cells, fibroblasts, and soluble proteins such as cytokines and growth factors.^20^, ^21^ Several cytokines, including interleukin (IL‐)1, IL‐6, IL‐8, and tumor necrosis factor‐alpha (TNF‐ɑ), have been shown to be associated with disease progression in PDAC.^22^, ^23^ A further investigation of inflammatory proteins as biomarkers in patients with PDAC is therefore an appealing next step in the quest for better biomarkers. Several attempts have been made in proteomics, but so far none have been translated to daily clinical practice.^24^, ^25^ One of the great challenges in proteomics is measuring low‐abundance disease‐associated proteins in a pool of abundant plasma proteins. The technology must be sensitive enough to reliably and accurately quantify the low‐abundant proteins in plasma.^26^ An increasingly used method for identifying multiple biomarkers in panels is the proximity‐extension assay (PEA). With this antibody‐based method, several panels of pre‐defined circulating proteins can be examined, e.g. the Immuno‐oncology (I‐O) panel from Olink Proteomics (Uppsala, Sweden, www.olink.com). We and others have demonstrated the use of this method in the diagnosis of PDAC.^27^, ^28^

In the present study, we used the I‐O protein panel from Olink to investigate 92 circulating proteins in patients eligible for surgery for suspected PDAC. Our hypothesis was that there was a panel of proteins that could distinguish patients with unresectable tumors from patients with resectable tumors, and further that there was a prognostic signature for the patients with resectable tumors.

## 2 MATERIALS AND METHODS

The study was conducted according to the REMARK (Reporting Recommendations for Tumor Marker Prognostic studies)^29^ and TRIPOD (Transparent Reporting of a multivariable prediction model for Individual Prognosis Or Diagnosis) guidelines.^30^

### 2.1 Patients

This prospective study included 273 patients undergoing surgery at the Department of Surgery, Copenhagen University Hospital—Rigshospitalet, Denmark, due to suspected PDAC. The patients were included in the BIOPAC study (“BIOmarkers in patients with PAncreatic Cancer (BIOPAC)—can they provide new information of the disease and improve diagnosis and prognosis of the patients”; ClinicalTrials.gov ID: NCT03311776; www.herlevhospital.dk/BIOPAC/) between July 2008 and November 2018. All patients provided written informed consent. The BIOPAC study protocol was approved by the regional ethics committee (VEK ref KA‐20060113) and the Danish Data Protection Agency (j.nr. 2012‐58‐0004; HGH‐2015‐027; I‐Suite j.nr. 03960; and PACTIUS P‐2020‐834). The study was conducted in accordance with the Declaration of Helsinki.

Clinical eligibility criteria were age >18 years and tentative diagnosis of potentially resectable PDAC. Potentially resectable pancreatic cancer was defined as a tumor limited to the pancreas with no arterial contact (superior mesenteric artery, celiac axis and common hepatic artery), and ≤180° contact with the superior mesenteric vein or portal vein without vein contour irregularity.^10^ Patients from the BIOPAC study with available samples were included in this study. Patients were followed until death or March 2, 2021. For patients alive at the cutoff date (*n* = 22), the median follow‐up time was 70 months.

### 2.2 Sample characteristics

Blood samples for analysis were collected within a median of 1 day (range 0–109 days) before surgery. In five patients, time from blood sampling to resection was >30 days (33, 36, 45, 46, 109 days). These patients are further described in the Supplement. All samples were centrifuged at 2300 *g* at 4°C for 10 min, and serum was then aliquoted in Greiner tubes (Cryo.s™ Freezing Tubes, 2 ml, GR‐121280, Greiner Bio‐One GmbH). The serum was subsequently stored at −80°C. Upon collection, samples were thawed at room temperature, mixed using a vortex mixer, and centrifuged at 1600 *g* for 10 min. Then, 250 μl was aliquoted to tubes (2.0 ml Graduated w/o Ribs Screw Tubes, Natural from SSIbio, CA, USA), labeled with an individual number, and stored at −80°C until analysis at BioXpedia.

### 2.3 CA19‐9

Serum levels of CA19‐9 were determined using the Immulite 2000 GI‐MA assay (Siemens, Catalog Number L2KG12), a solid‐phase, two‐site sequential chemiluminescent immunometric assay. Imprecision was monitored with two internal controls at 16 and 83 U/ml with coefficients of variation of 8% and 9%. Accuracy was monitored within the standard UK NEQAS program. Elevated CA19‐9 was defined as >37 U/ml.

### 2.4 Olink immuno‐oncology assay

Using the Olink Immuno‐Oncology assay, serum samples were analyzed for 92 proteins (for full list of proteins, see Table S1). Using the PEA technology, 1 μl of serum sample was mixed with a set of 92 pairs of antibodies linked to oligonucleotides (probes). After binding to the target antigen, the probes are brought into proximity, which leads to the oligonucleotides being extended by a DNA polymerase. This acts as a surrogate marker for the specific antigen and is amplified and quantified by real‐time polymerase chain reaction (qPCR), where the number of PCR templates formed is proportional to the initial concentration of antigen in the sample.^31^, ^32^ The resulting abundance level for each protein is given as NPX (Normalized Protein eXpression) values on a log2 scale. Assay characteristics including detection limits calculations, assay performance, and validations are available from the manufacturer (www.olink.com). The assay was performed at BioXpedia, Aarhus, Denmark, according to the manufacturer's instructions and was done blinded to the study endpoints.

### 2.5 Statistical analyses

This was an exploratory biomarker study, and therefore no power calculations for appropriate study size could be made.

The same data and predefined plan for analyses were given to two independent bioinformaticians. Differential expression of circulating proteins between patient groups (see below) was tested using a *t*‐test for independent samples. If the central limit theorem did not apply because the groups of interest had less than 40 samples, we checked whether the two tested groups were normally distributed using the Shapiro–Wilk test. If one group was not normally distributed, a Wilcoxon rank sum test was conducted instead. Furthermore, the fold change was calculated on a linear scale as the geometric mean of the first group divided by the geometric mean of the second group. The *p*‐values were corrected for multiple testing using the Benjamini‐Hochberg method.^33^ *p*‐values of <0.05 were considered statistically significant.

In the first statistical approach, the 92 proteins of the Olink panel plus CA19‐9 were tested for differential expression in the following comparisons: resectable versus unresectable; resectable with (i) overall survival (OS) <1 year versus >1 year, (ii) OS <1 years versus >3 years, (iii) OS <1 years versus > 4 years, (iv) OS <1 years versus >5 years; unresectable with OS <median versus >median; resectable with OS <median versus >median. Volcano plots were generated to illustrate the relationship between the *p*‐values and fold changes for the 93 markers in each of the comparisons. Boxplots were created for the proteins with unadjusted significant *p*‐values.

A principal component analysis was carried out in all samples grouped into resectable with OS <1 year, resectable with OS >1 year, and unresectable.

Both bioinformaticians were also asked to identify a panel of proteins for prognostic purposes. In the first statistical approach, a two‐step strategy was employed. In short, the first step explored the performance and stability of the complete set of proteins as predictors in logistic elastic‐net (LASSO and Ridge) regression models.^34^ Samples were divided into discovery and replication cohorts, and the discovery cohort was further split into two equally large parts, thus generating a training set and a test set. Using the complete set of differentially expressed proteins, a logistic LASSO regression model was fitted on the training set using the R‐package *glmnet* with *alpha* = 1 and optimized with the function *cv*.*glmnet* using 10‐fold cross validation in the training set. The fitted logistic LASSO regression model was then employed on the test set. This process was repeated 500 times, thus generating 500 different logistic LASSO regression models. For each protein it was noted how many times each of the 500 logistic LASSO regression models included that protein as a predictor. These values were noted as a proportion and therefore were referenced to as the proportion score. The proteins with the highest proportion score were taken as the most stable predictors and the proteins with the lowest proportion scores were taken as the least stable predictors. To identify protein signatures containing the most stable proteins, 21 sets of proteins were generated according to the proportion scores. The sets were constructed so that the first set contained proteins with a minimum proportion score of 0 and thus contained all differentially expressed proteins. The remaining 20 sets were constructed according to an incremental step of the proportion score of 0.05. For instance, the second set contained proteins that had a minimum proportion score of 0.05 (included in at least 5% of the models) and the 21st and final set contained proteins with a minimum proportion score of 1. For sets of proteins that were identical, only the sets with the corresponding highest proportion score were selected and therefore some of the incremental steps were skipped.

The second step involved finding the best performing protein signatures from step one by evaluating the performance of the protein signatures in the discovery and replication cohorts.

For each protein signature, a Ridge regression model was fitted on the training set of the discovery cohort and tested in the corresponding test set using the R‐package *glmnet* with *alpha* = 0 and optimized with the function *cv*.*glmnet* using 10‐fold cross validation in the training set. The same procedure was carried out where the entire discovery cohort was used as a training set and the entire replication cohort was used as a test set. The performance of each of the generated models was evaluated using receiver operating characteristics (ROC) curves and the area under the curve (AUC). For each ROC curve, Youden's index, also referred to as the best point, was used to identify the cutoff with the highest sensitivity and specificity. The robustness of all evaluation parameters (AUC, sensitivity, specificity, positive predictive value [PPV], and negative predictive value [NPV]) was investigated using bootstrapped 95% confidence intervals (CI) and 2000 stratified bootstrap replicates with the R package *pROC* version 1.16.2. All statistical analyses used in the first statistical approach were made using R version 4.0.2.

In the second statistical approach, the patients were not divided into discovery and replication cohorts. Comparison of protein levels between the groups was made with Wilcoxon rank sum test. For each protein, univariate and multivariate Cox proportional hazards models, including age, stage, ASA score (American Society of Anesthesiologists Physical Status Classification System), CA19‐9 level, type of adjuvant chemotherapy, were used to determine its effect on OS in both the cohort of all patients and the group of resectable patients. Kaplan–Meier curves were used to plot the results and logrank *p*‐values were determined. After standardization of the protein levels, the L1 (LASSO)‐regularized logistic regression model was trained to separate the resectable and unresectable tumors.^34^ LASSO regularization was chosen due to its performance with data with high collinearity, and due to its inherent variable selection. The model was trained using the *glmnet* R package. The penalty parameter (*λ*) was trained using a tenfold nested cross‐validation strategy. For a robust solution in both lambda and the weights, this 10‐fold cross‐validation step was repeated 500 times. The final parameter *λ* was selected as the median of the resulting 500 *λ* values, and the final model weights were achieved using this value. The distribution of the resulting weights from the 500 iterations signifies the robustness of the final model.

The LASSO‐regularized Cox regression was used to predict OS. The steps in the model‐building were similar to the LASSO‐regularized logistic regression described above. For model‐building, our aim was to develop a classifier that could differentiate patients with resectable tumors with an OS >3 years from patients with an OS <1 year and a prognostic model for all patients and patients with non‐resectable tumors. A prediction score for each patient was calculated using the linear combination of levels of each protein multiplied by the LASSO coefficient. For the evaluation of the model, ROC curves were created using the *pROC* R package and the AUCs were calculated. Time‐dependent ROC curves were made using the *survivalROC* R package, and the survival of patients with high or low risk scores was compared using Kaplan–Meier and Cox proportional hazards models. High or low risk score groups were defined either by median risk score value or the best cutoff using *maxstat* R package. The prediction score was compared to the survival status at 6, 12, and 24 months. True positive meant that the patient was predicted to be alive and was in fact alive at that timepoint. Just as we did when we tried to separate and predict patients with long versus short survival, a non‐negative L1 (LASSO)‐regularized Cox Regression model was trained to predict overall survival (family = “cox” in *glmnet*). The risk score was evaluated using ROC, time‐dependent ROC, and survival curves to determine median and best cutoff for the risk score. All statistical analyses used in the second statistical approach were made using the R version 3.6.3.

## 3 RESULTS

Of the 273 patients, 193 patients had resectable tumors, leaving 80 patients with unresectable tumors. Of the patients with resectable tumors, 111 (57.5%) underwent a pancreatico‐duodenectomy, 28 (14.5%) distal pancreatectomy, and 54 (28.0%) total pancreatectomy. The relatively high numbers of total pancreatectomies may reflect that all borderline resectable tumors were operated, and pre‐operative chemotherapy was very seldomly used in the time period when the patients were included (2008–2018). The standard was to remove the entire pancreas if a tumor in the caput of pancreas stretches to the left of the mesenteric blood vessels, if resection margins are unclear, or if pancreatic anastomosis is uncertain with a risk of pancreatic leak, and the patient has type 1 diabetes mellitus and only a small remaining pancreas. The number of patients with borderline resectable tumors was not recorded because both patients with localized and borderline resectable tumors were treated with up‐front surgery in the time period 2008–2018.

Of the patients with unresectable tumors, 22 (27.5%) were found to have locally advanced disease, and 58 (72.5%) had metastatic disease. Of the 273 patients, 12 (4.4%) patients had received any chemotherapy prior to surgery. Of these 12 patients, eight patients achieved resection.

For further patient characteristics, see Table 1.

**TABLE 1:** Patient characteristics before surgery

	No. (%)^a^ of patients					
	Unresectable	Resectable				Total number of patients
		Resected, short‐term survivors (<1 year)	Resected, survival 1–3 years	Resected, long‐term survivors (>3 years)	All resected	
Number of patients	80 (29.3)	64 (23.4)	70 (25.7)	59 (21.6)	193 (70.7)	273 (100)
Sex						
Men	43 (53.8)	35 (54.7)	39 (55.7)	32 (54.2)	106 (54.9)	149 (54.6)
Women	37 (46.2)	29 (45.3)	31 (44.3)	27 (45.8)	87 (45.1)	124 (45.4)
Age, median (range)	67 (40–84)	68 (38–86)	66 (38–81)	65 (37–80)	67 (37–86)	67 (37–86)
>70 years, *n* (%)	25 (31.3)	24 (37.5)	23 (32.9)	13 (22.0)	60 (31.1)	85 (31.1)
Stage I	—	2 (3.1)	2 (2.9)	8 (13.6)	12 (6.2)	12 (4.4)
Stage II	—	29 (45.3)	38 (54.3)	32 (54.2)	99 (51.3)	99 (36.3)
Stage III	22 (27.5)	32 (50.0)	30 (42.8)	18 (30.5)	80 (41.5)	102 (37.3)
Stage IV	58 (72.5)	1 (1.6)	—	1 (1.7)	2 (1.0)	60 (22.0)
Type of surgery, pancreaticoduodenectomy	—	34 (53.1)	42 (60.0)	35 (59.3)	111 (57.5)	111 (40.7)
Distal pancreatectomy	—	5 (7.8)	10 (14.3)	13 (22.0)	28 (14.5)	28 (10.2)
Total pancreatectomy	—	25 (39.1)	18 (25.7)	11 (18.7)	54 (28.0)	54 (19.8)
Exploratory laparotomy	80 (100)	—	—	—	—	80 (29.3)
ASA score I	21 (26.3)	16 (25.0)	18 (25.7)	19 (32.2)	53 (27.5)	74 (27.1)
ASA score II	38 (47.5)	24 (37.5)	41 (58.6)	32 (54.2)	97 (50.2)	135 (49.4)
ASA score III	20 (25.0)	24 (37.5)	11 (15.7)	7 (11.9)	42 (21.8)	62 (22.7)
ASA score IV	1 (1.2)	—	—	—	—	1 (0.4)
ASA score unknown	—	—	—	1 (1.7)	1 (0.5)	1 (0.4)
BMI, <18.5	6 (7.5)	4 (6.3)	5 (7.2)	—	9 (4.7)	15 (5.5)
18.5–25	39 (48.7)	36 (56.2)	39 (55.7)	36 (61.0)	111 (57.5)	150 (54.9)
>25	34 (42.5)	22 (34.4)	25 (35.7)	23 (39.0)	70 (36.3)	104 (38.1)
Unknown	1 (1.3)	2 (3.1)	1 (1.4)	—	3 (1.5)	4 (1.5)
Diabetes at diagnosis	20 (25.0)	26 (40.6)	17 (24.3)	14 (23.7)	57 (29.5)	77 (28.2)
Tobacco, Never smoker	19 (23.8)	20 (31.3)	27 (38.6)	26 (44.1)	73 (37.8)	92 (33.7)
Former smoker	29 (36.2)	21 (32.8)	24 (34.3)	17 (28.8)	62 (32.1)	91 (33.3)
Current smoker	32 (40.0)	23 (35.9)	19 (27.1)	16 (27.1)	58 (30.1)	90 (33.0)
Alcohol, within recommendations^b^	55 (68.8)	54 (84.4)	49 (70.0)	46 (78.0)	149 (77.2)	204 (74.7)
Over recommendations^b^	12 (15.0)	5 (7.8)	15 (21.4)	7 (11.8)	27 (14.0)	39 (14.3)
Former overuse	13 (16.2)	4 (6.3)	6 (8.6)	6 (10.2)	16 (8.3)	29 (10.6)
Unknown alcohol consumption	—	1 (1.5)	—	—	1 (0.5)	1 (0.4)
CA19‐9, U/ml, median (IQR)	330 (50–1025)	347 (127–1085)	169 (38–851)	88 (26–282)	169 (43–740)	207 (49–886)
Overall survival, months, median (IQR)	8.2 (5.1–13.8)	7.8 (5.5–9.9)	23.1 (16.8–30.4)	61.0 (47.3–75.6)	22.6 (9.9–45.3)	15.2 (7.7–34.4)

*Note*: Definition of ASA scores; I: normal health, II: mild systemic disease, III: severe systemic disease, IV: severe systemic disease that is a constant threat to life.

Abbreviations: ASA score, American Society of Anesthesiologists Physical Status Classification System; BMI, body mass index; IQR, Interquartile range.

^a^ Unless otherwise specified.

^b^ The recommended alcohol intake is maximum 84 g/week for women and maximum 168 g/week for men (The Danish Health Authorities).

Of patients with resectable tumors, one had a metastasis in a non‐regional lymph node (OS 52.1 months), and one patient had liver metastases on a CT scan 2 months after surgery (OS 7.7 months).

Of patients with resectable tumors, 64 were “short‐term survivors” (<1 year), 70 patients had an OS between 1 and 3 years (1 patient censured at end of follow‐up), and 59 patients were “long‐term survivors” with an OS of >3 years (6 patients censured at end of follow‐up). For an overview of the patient flow in the two statistical approaches, see Figure S1.

Each of the 92 proteins in the I‐O panel were compared with survival in the entire patient cohort. For each protein, patients with resectable tumors were divided according to NPX levels (< median or > median). Kaplan–Meier curves of the proteins with logrank *p*‐values <0.05 are shown in Figure S2.

### 3.1 Differentiation between patients with resectable and non‐resectable tumors

Proteins with statistically significant *p*‐values (unadjusted) are shown in Table S2 and visualized in volcano plots (Figure S3) and boxplots (Figure S4). No *p*‐values were significant after adjusting for multiple testing (Table S3).

We also sought to identify a panel of circulating proteins for the identification of patients who would benefit from surgery. In the first statistical approach, a principal component analysis was performed according to protein levels. However, the two groups did not separate clearly, see Figure S5. Therefore, no further model‐building was performed using this approach.

In the second statistical approach, the analysis was different (see Methods), and the model‐ building was pushed forward, resulting in a signature with six proteins, for details see Table 2. This protein signature had an AUC value of 0.66 (95% CI 0.59–0.73). The ROC plot and the weights for the included proteins are found in Figure S6.

**TABLE 2:** All proteins included in the models with weights, AUCs, sensitivity, and specificity

Protein	Weights in the protein signatures						
	Resectable versus unresectable	Prognostic panel for resectable patients		Resectable patients: OS <1 year versus OS >3 years		Prognostic panel for unresectable patients	
		Index I	Index II	Index III	Index IV	Index V	Index VI
ADA	0.0472037	—	—	—	—	—	—
ADGRG1	—	—	—	—	—	−0.6637	—
CAIX	—	−0.25995	—	—	—	—	—
CCL20	—	—	—	—	—	—	0.2980801
CD5	0.0751803	—	—	—	—	—	00674279
CD8A	—	—	—	—	—	—	0.0076804
CD27	—	−0.29162	—	—	—	—	—
CD40	—	—	0.0058470	—	—	—	—
**CD40L**	—	0.124044	—	0.484579	—	—	—
CD244	0.0570681	—	—	—	—	—	—
**CXCL1**	—	—	0.0010794	—	0.0086580	—	—
CXCL9	—	—	—	0.187991	—	—	—
FGF2	—	—	—	—	—	0.574169	—
GZMB	—	0.217839	—	—	—	—	—
GZMH	—	—	—	0.195087	—	—	—
IFN‐gamma	—	0.1595	—	—	—	—	—
IL‐1 alpha	—	—	—	−0.20561	—	—	—
**IL‐4**	—	—	0.0150466	—	−0.0089399	—	—
**IL‐5**	—	0.214107	—	−0.26109	—	—	—
**IL‐6**	—	—	0.0727595	0.282344	0.1156411	—	0.0700218
IL‐10	—	—	—	—	—	0.495288	—
IL‐12	0.0366291	—	—	—	—	—	—
IL‐33	—	0.231262	—	—	—	—	—
MIC‐A/B	0.0395936	—	—	—	—	—	—
**PTN**	—	−0.35061	—	—	—	0.561139	—
VEGF‐C	0.0950826	—	—	—	—	—	—
VEGFR‐2	—	—	—	−0.28793	—	—	—
CA19‐9	—	—	—	0.293196	—	—	—
intercept	0.8895062	0.662921	—	0.01126	—	0.763352	—
AUC values, sensitivity, and specificity of the indices							
AUC, (95%CI)	0.66 (0.59–0.73)	—	0.50–0.55*	—	0.53–0.55*	—	0.71–0.89*
AUC, discovery (95% CI)	—	0.75 (0.60–0.90)	—	0.74 (0.57–0.91)	—	0.78 (0.56–1.0)	
AUC, replication (95% CI)	—	0.62 (0.46–0.79)	—	0.63 (0.47–0.79)	—	0.64 (0.42–0.86)	
Sensitivity (95% CI)	—	D: 0.83 (0.59–1.0) R: 0.43 (0.24–1.0)	—	D: 0.72 (0.50–0.94) R: 0.55 (0.18–0.88)	—	D: 1.0 (0.66–1.0) R: 0.65 (0.25–1.0)	
Specificity (95% CI)	—	D: 0.68 (0.36–0.89) R: 0.83 (0.25–1.0)	—	D: 0.84 (0.68–1.0) R: 0.73 (0.39–1.0)	—	D: 0.60 (0.40–1.0) R: 0.77 (0.33–1.0)	

*Note*: Proteins in **bold** are included in >1 index.

Abbreviations: D, Discovery cohort, R, Replication cohort.

* Range of AUC‐values from time‐dependent ROC plots.

### 3.2 Prognostic panel for patients with resectable tumors

**FIGURE 1:**
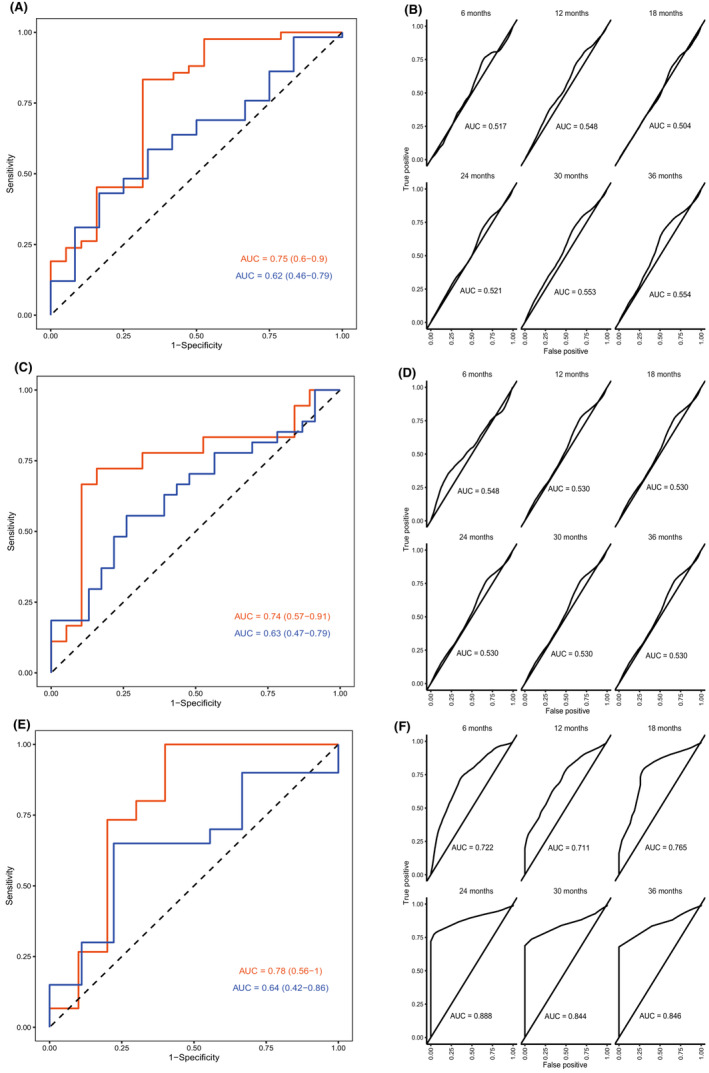
Performance of Index I–VI. (A) Index I and Index II, predicting survival of all patients with resectable tumors, median OS (22.6 months) used as cutoff. The plot with two curves represents Index I, and the time‐dependent ROC curves represent Index II. (B) Index III and Index IV, predicting short (<1 year, *n* = 64) versus long survival (>3 years, *n* = 59) of patients with resectable tumors. The plot with two curves represents Index III, and the time‐dependent ROC curves represent Index IV. (C) Index V and Index VI, predicting survival of all patients with unresectable tumors, median OS (8.2 months) used as cutoff. The plot with two curves represents Index V, and the time‐dependent ROC curves represent Index VI. For Indices I, III, and V, the orange curve represents the discovery cohort, and the blue curve represents the replication cohort. For Indices II, IV, and VI, the time‐dependent ROC curves represent the performances of the indices at 6, 12, 18, 24, 30, and 36 months.

We then examined prognostic protein panels for predicting survival in the group of resected patients (*n* = 193) using the median OS (22.6 months) of this group as a cutoff. With the first statistical approach, five signatures were found (Table 3, A1–A5). The signature with eight proteins performed best, for details see Table S4. This signature, named Index I, had an AUC of 0.75 (95% CI 0.60–0.90) in the discovery cohort and 0.62 (0.46–0.79) in the replication cohort with a sensitivity of 0.83 (0.59–1.0) and 0.43 (0.24–1.0), respectively, and a specificity of 0.68 (0.36–0.89) and 0.83 (0.25–1.0), respectively. The performance of this signature is visually presented as a ROC plot in Figure 1A, and the weights of included proteins are found in Table 2.

**TABLE 3:** Candidate prognostic plasma protein signatures from the following comparisons: (A) Resectable with OS <1 year versus >3 years, (B) Resectable with OS < median versus > median (22.6 months), and (C) Unresectable with OS < median versus > median (8.2 months)

	Resectable					Resectable						Unresectable				
	OS < median versus > median					OS <1 year versus > 3 years						OS < median versus > median				
Abbreviated protein names	A1	A2	A3	A4	A5	B1	B2	B3	B4	B5	B6	C1	C2	C3	C4	C5
ADGRG1	All 93 markers					All 93 markers						All 93 markers	X	X		
ARG1													X			
CAIX		X	X	X												
CCL17													X			
CD5													X			
CD27		X	X													
CD40							X									
CD40‐L		X	X				X	X	X	X						
CXCL9							X	X								
CXCL11							X									
CXCL12							X									
CXCL13							X									
FASLG							X									
FGF2		X					X						X	X	X	X
GZMB		X	X													
GZMH							X	X	X							
IFN‐beta							X									
IFN‐gamma		X	X				X						X			
IL‐1‐alpha							X	X								
IL‐4							X									
IL‐5		X	X	X			X	X								
IL‐6							X	X								
IL‐7							X									
IL‐10													X	X		
IL‐12							X									
IL‐13		X														
IL‐33		X	X				X									
IL‐35		X					X									
PDGF subunit‐B							X									
PTN		X	X	X	X		X						X	X	X	
TNFSF9													X			
TNFSF14													X			
TRAIL													X			
TWEAK							X									
VEGFR‐2							X	X	X							
CA19‐9							X	X	X	X	X					
**Proteins, *n***	**93**	**11**	**8**	**3**	**1**	**93**	**24**	**8**	**4**	**2**	**1**	**93**	**11**	**4**	**2**	**1**
**Proportion score**	**0**	**0.05**	**0.1**	**0.15**	**0.5**	**0**	**0.05**	**0.1**	**0.15**	**0.2**	**0.25**	**0**	**0.05**	**0.1**	**0.15**	**0.2**

**FIGURE 2:**
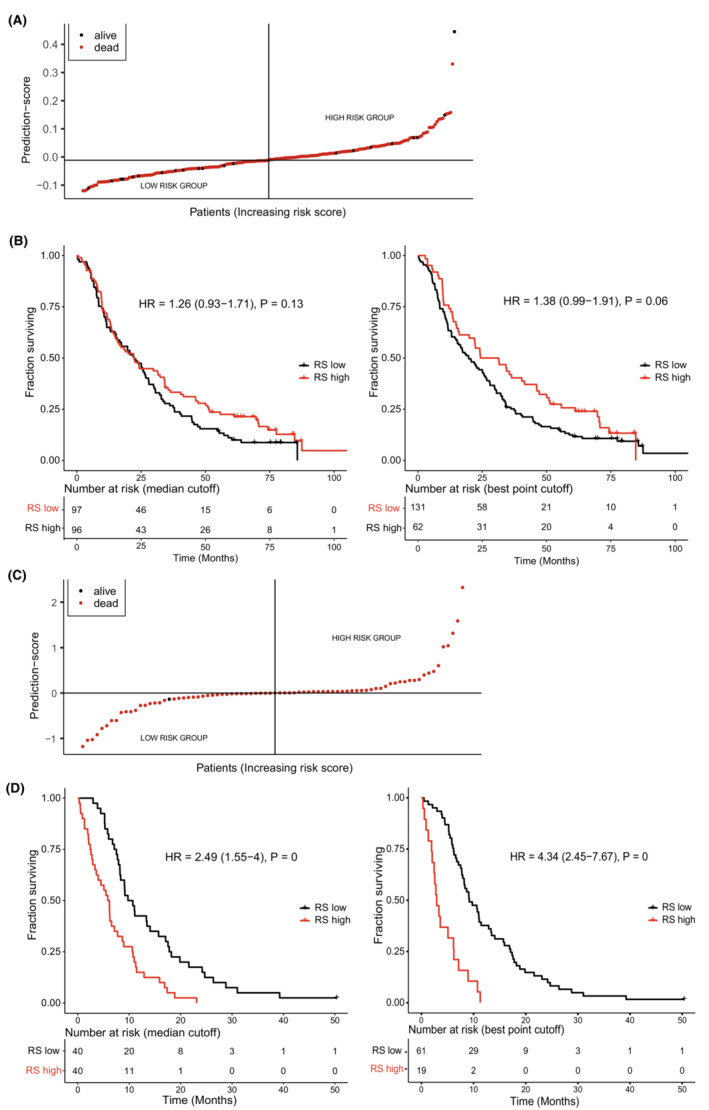
Prediction score versus survival for resectable patients (Index IV) and for unresectable patients (Index VI). (A) Index IV, distribution of patients according to prediction score of survival for patients with resectable tumors. (B) Index IV, prediction score versus survival, Kaplan–Meier plot, median risk score cutoff (left), and best point risk score cutoff (right) for resected patients. (C) Index VI, distribution of patients according to prediction score of survival for patients with unresectable tumors. (D) Index VI, prediction score versus survival, Kaplan–Meier plot, median risk score cutoff (left), and best point risk score cutoff (right) for un‐resected patients.

With the second statistical approach, one signature was found that contained four proteins (Index II). Time‐dependent ROC plots comparing prediction score and survival status are shown in Figure 1A. Plots for the prediction score, including a Kaplan–Meier analysis of patients divided by low or high prediction score, are found in Figure 2. No proteins overlapped between Index I and Index II.

For each individual I‐O protein, Kaplan–Meier plots were made for the group of resectable patients, divided by NPX levels (< median or > median). Proteins with logrank *p*‐values <0.05 are shown in Figure S2.

### 3.3 Patients with resectable tumors: short‐term survivors and long‐term survivors

Among the 193 patients with resectable tumors, 64 patients had short‐term survival (<1 year) and 59 patients long‐term survival (>3 years). The first statistical approach revealed six signatures (Table 3), with the best performing signature containing eight proteins (Index III). For details, see Table 2. This signature had an AUC value of 0.74 (95% CI 0.57–0.91), sensitivity of 0.72 (0.50–0.94), and a specificity of 0.84 (0.68–1.0) in the discovery cohort. In the replication cohort the AUC was 0.63 (0.47–0.79), sensitivity 0.55 (0.18–0.88), and specificity 0.73 (0.39–1.0). Details of the performance of the other five candidate signatures are shown in Table S4 (signatures B1‐B6). A ROC plot showing the performance of Index III can be found in Figure 1B.

With the second statistical approach, one signature was found containing three proteins (Index IV). Only IL‐6 overlapped between Index III and IV. Time‐dependent ROC plots for Index IV that compare prediction score for survival status are shown in Figure 1B. Plots for the prediction score, including Kaplan–Meier analysis of patients divided by low or high prediction score, are found in Figure S7.

### 3.4 Prognostic panel for patients with unresectable tumors

Patients with unresectable tumors (*n* = 80) were also analyzed as a subgroup. We examined whether panels of proteins could predict whether a patient would survive longer or shorter than the median OS in this subgroup (8.2 months). The first statistical approach found five signatures, where the signature with four proteins had the best performance. For performance of all candidate signatures, see Table 3 (C1–C5). This signature (Index V) had an AUC of 0.78 (95% CI 0.56–1.0) in the discovery cohort and 0.64 (0.42–0.86) in the replication cohort. Sensitivity was 1.0 (0.66–1.0) in the discovery cohort and 0.65 (0.25–1.0) in the replication cohort. Specificity was 0.60 (0.40–1.0) and 0.77 (0.33–1.0) in the discovery and replication cohorts, respectively. For a ROC plot, see Figure 1C.

Using the second statistical approach, one signature was found, which also included four proteins (Index VI). However, no proteins overlapped between Index V and Index VI, see Table 2.

Time‐dependent ROC plots of Index VI are shown in Figure 1C. Plots for the prediction score, including Kaplan–Meier analysis of patients divided by low or high prediction score, are found in Figure 2.

All proteins included in the models are shown with weights and with AUC values, sensitivity, and specificity where available (see Table 2).

## 4 DISCUSSION

Identifying the patients with PDAC eligible for resection is challenging, and better biomarkers are needed to improve this selection. Here, we attempted to find panels of proteins that could be used for this selection and for prognostic use in subgroups of patients with PDAC. However, none of the models performed well enough to warrant further exploration.

The method (PEA) used for analyzing serum samples in our study has previously been shown to be applicable for diagnostic use in patients with PDAC.^27^, ^28^ In a previously published study by our group,^27^ protein levels in patients with PDAC (stage I–IV) were compared with levels in patients with non‐malignant pancreatic diseases and healthy individuals. We included 701 patients with PDAC, 102 patients with non‐malignant pancreatic diseases, and 180 healthy blood donors. In that study, we identified two protein indices for the identification of patients with PDAC, both with performances reaching AUC values of ≥0.92.^27^ The patients included in the present study were also a part of the cohort in the previously published study.

We had hypothesized that the PEA platform was applicable in the setting of patients with potentially resectable PDAC tumors, and that it would be possible to separate patients with regard to resectability according to the levels of I‐O proteins. This proved not to be the case. In a recently published study, several systemic inflammatory markers, including the neutrophile‐to‐lymphocyte ratio (NLR), were evaluated in patients with metastatic PDAC, and all markers were significantly associated with OS.^35^ A meta‐analysis also found that a high NLR correlated with tumor metastasis.^36^ Thus, we had expected that increased inflammation would be found in patients with unresectable tumors, especially in the 58 patients with metastatic disease, as inflammation is known to promote cancer progression and metastasis.^18^, ^19^

Evaluation of resectability in PDAC may be difficult, and between 20 and 40% of patients deemed eligible for resection are found to have locally advanced or metastatic disease during surgery.^8^, ^9^ Even though the quality of the pre‐operative imaging and the qualifications of the radiologist may be improved in a number of cases, metastatic disease may not be detectable, neither before nor during surgery. Patients who develop metastases within a few months after expected radical surgery constitute a well‐known and unfortunate problem, regardless of the use of guidelines for resection and neoadjuvant chemotherapy. Therefore, it would be of great value to have a biomarker panel that could aid in the decision to operate or not.

In our study, the protein indices for prognostic use in resected patients had low AUC values between 0.50 and 0.75. For patients with unresectable tumors, the highest AUC value was achieved with Index VI, the time‐dependent ROC at 24 months having an AUC of 0.89. This may serve as a prognostic tool for the identification of patients with a favorable prognosis in a group where the median OS is less than 1 year.^37^, ^38^, ^39^

The statistical approaches and strategies used to identify the proteins included in our indices differed, and few proteins overlapped between the indices. Only one protein, IL‐6, was included in >2 indices. IL‐6 is a known prognostic marker in both local and metastatic pancreatic cancer.^23^, ^40^, ^41^ IL‐6 plays an important role in inflammation and leads to a secretion of both pro‐inflammatory and immunosuppressive factors in tumor cells and to the promotion of anti‐apoptosis, angiogenesis, and invasion.^42^ The fact that IL‐6 is included in several of our indices, identified through use of two different statistical methods, speaks for the validity of our results. Few other proteins overlapped between our indices, and they can be grouped as immune stimulating (CD40 and CD40L^43^), angiogenic and tumor promoting (CXCL1^44^ and PTN^45^), and immune suppressing (IL‐4^46^ and IL‐5^47^).

CA19‐9 is the most well‐studied biomarker in pancreatic cancer, with a sensitivity of 79% and a specificity of 82%.^48^ However, the biomarker has a number of short comings as mentioned above, which makes its use as a diagnostic marker less valid, while its use in monitoring oncologic treatment is more reliable.^13^, ^14^, ^15^ In borderline resectable tumors evaluated by CT scan, an elevated CA 19–9 together with confirmed lymph node metastases results in a recommendation to use neoadjuvant chemotherapy rather than undertake upfront resection.^49^ In our study, CA19‐9 survived the selection procedures only for Index III.

Other biomarkers have been studied to determine resectability and prognosis for patients with potentially resectable PDAC. In a recent study including 290 patients with resectable PDAC from the BIOPAC study, a combination of CA19‐9, IL‐6 (determined by ELISA) and YKL‐40 (also known as chitinase 3‐like 1, CHI3LI) showed potential for preoperatively identifying a subgroup of patients with poor survival.^23^ Patients with levels of all three biomarkers above the median had significantly reduced OS compared with patients with low levels of the three markers.^23^ High IL‐6 alone was also associated with unresectability and a poor OS for the patients with resectable PDAC.^23^

Another study in patients from the BIOPAC study analyzed CRP, CA19‐9, IL‐6 (determined by ELISA), and YKL‐40 in 993 patients with PDAC independent of stage.^41^ Here, increasing IL‐6, CA19‐9, CRP, and YKL‐40 were independently associated with poor survival in a cohort where 30% had resectable PDAC.^41^ In the present study, no association was found between IL‐6 in patients with resectable and unresectable tumors. However, high IL‐6 was associated with poor survival in the patients with resectable tumors as well as in all patients in combination (resectable and unresectable).

The data obtained in the present study can be utilized as a resource to initiate novel research lines focusing on the disease relevance of individual proteins of interest, rather than on protein signatures since these clearly are not clinically relevant in this context.

Our study has several strengths and limitations. Two bioinformaticians worked independently of one another on our data, which strengthens the study, because there is no established statistical method for this type of biomarker discovery studies. However, this also highlights the variability, as few proteins overlapped between the indices found by the two statistical strategies. This can be explained by the different statistical approaches used, e.g. one dividing patients into discovery and replication cohorts, the other not doing so; one choosing ROC plots and AUC values with sensitivity and specificity, the other choosing time‐dependent ROCs and a risk score. One method is not to be preferred over the other, but this potentially increases the variability and makes it more difficult to directly compare two sets of results. Another explanation is that the results are somewhat random since little actual effect or correlation exists between the examined I‐O proteins and the hypotheses in question. Yet another explanation is the sample size. Even though we included nearly 300 patients, the division into several groups according to resection and survival rendered some of the groups quite small.

Furthermore, when doing multiple testing, as we did when exploring 93 markers in different groups, adjusting for this statistically is necessary. However, when adjusting for multiple testing in a quest to reduce false positives, there may be an increase in false negatives, meaning we might miss some of the proteins that are actually differentially expressed.

In conclusion, our study did not find biomarker indices with sufficient performance to aid in the identification of patients with resectable PDAC. Nor were we able to identify prognostic biomarkers superior to already existing biomarkers. The Olink immuno‐oncology panel has been shown to yield interesting new results regarding the diagnosis of PDAC but does not seem to need further investigation regarding the identification of patients with resectable PDAC.

## AUTHOR CONTRIBUTIONS

**Sidsel Christy Lindgaard:** Conceptualization (equal); formal analysis (supporting); funding acquisition (equal); investigation (lead); methodology (equal); project administration (lead); resources (equal); visualization (equal); writing – original draft (lead); writing – review and editing (equal). **Zsófia Sztupinszki:** Formal analysis (equal); methodology (equal); software (equal); visualization (equal); writing – review and editing (equal). **Emil Maag:** Formal analysis (equal); methodology (equal); software (equal); visualization (equal); writing – review and editing (equal). **Carsten P. Hansen:** Resources (equal); writing – review and editing (equal). **Inna Chen:** Conceptualization (equal); supervision (equal); writing – review and editing (equal). **Astrid Z. Johansen:** Resources (equal); writing – review and editing (equal). **Jane P. Hasselby:** Resources (equal); writing – review and editing (equal). **Stig E Bojesen:** Resources (equal); writing – review and editing (equal). **Dorte Nielsen:** Conceptualization (equal); supervision (equal); writing – review and editing (equal). **Julia S. Johansen:** Conceptualization (equal); funding acquisition (equal); resources (equal); supervision (equal); writing – review and editing (equal).

## FUNDING INFORMATION

This study received funding from The Beckett Foundation, Foundation of Merchant M. Kristjan Kjær and wife Margrethe Kjær, Memorial Fund of Carpenter Holm, Celgene, VELUX Foundation, Harboe Foundation, and the Danish Cancer Society.

## CONFLICT OF INTEREST

The authors declare no potential conflicts of interest.

## ETHICS APPROVAL STATEMENT

The BIOPAC study protocol was approved by the regional ethics committee (VEK ref KA‐20060113) and the Danish Data Protection Agency (j.nr. 2012‐58‐0004; HGH‐2015‐027; I‐Suite j.nr. 03960; and PACTIUS P‐2020‐834).

## Supporting information


Appendix S1

## Data Availability

The data that support the findings of this study are available from the corresponding author upon reasonable request.
