# Supplementary material for: Prognostic value of circulating proteins in patients undergoing surgery for pancreatic cancer
Source: Cancer Med. 2022 Oct 17;12(4):3972–86. doi: 10.1002/cam4.5240 (PMC9972037; doi:10.1002/cam4.5240)
Supplement: Supplementary file 1 — Appendix S1 [file CAM4-12-3972-s001.docx]

# Supplement: “Prognostic value of circulating proteins in patients undergoing surgery for pancreatic cancer”

**Supplementary methods**

**REMARK guidelines checklist**

**TRIPOD guidelines checklist**

**Supplementary Table S1:** Full list of proteins in the immuno-oncology panel.

**Supplementary Table S2**: Differential expressions of proteins between groups, only significant P-values given (unadjusted).

**Supplementary Table S3:** All tests run to determine differential expressions of proteins between groups.

**Supplementary Table S4:** Performance of the prognostic plasma protein signatures from the comparisons: Resectable with OS <1 year vs. >3 years (A1-6), Resectable with OS <median vs. >median (B1-5), and Unresectable with OS <median vs. >median (C1-5).

**Supplementary Table S5:** Mean NPX values of proteins included in all our protein signatures compared to NPX values for the misclassified patients.

**Supplementary Figure S1:** Patient flow for the two statistical approaches.

**Supplementary Figure S2:** Kaplan-Meier curves of proteins with *P* < 0.05. Divided according to NPX values (<median or >median).

**Supplementary Figure S3:** Volcano plots using unadjusted *P*-values.

**Supplementary Figure S4:** Boxplots with differential expressions of proteins with significant *P*-values (unadjusted).

**Supplementary Figure S5:** Principal component analyses dividing patients according to resection.

**Supplementary Figure S6:** ROC plot for differentiation between resectable and unresectable patients.

**Supplementary Figure S7:** Prediction score vs. survival for Index IV.

# Supplementary methods

## Patients

Patients were chosen from the BIOPAC study.

The five patients where time from blood sample to resection was >30 days are described here. For four of the patients, time between blood sample and surgery was 33–46 days. One patient had surgery postponed due to dehydration requiring a short hospital admission. One patient had a case of severe hypotension during an exploratory laparotomy, leading to a discontinuation of the planned resection and postponement of the resection to a later date. During the wait, the patients were examined by a cardiologist, and some of their antihypertensive medications were discontinued. In two patients, the resections were delayed slightly due to administrative problems.

The fifth patient, with 109 days between blood sample and resection, was misclassified by mistake. This patient should have been classified as unresectable but was mistakenly classified as resectable in the analyses. The patient was admitted to the department of surgery, where an exploratory laparotomy was performed. The patient was found to have an unresectable tumor that had spread into the superior mesenteric vein. After 2 months of chemotherapy, resection was tried again, and the patient had the tumor removed. The blood sample was taken prior to the first attempted resection. The protein levels of this patient are shown with the mean of both resectable and unresectable patients in Supplementary Table S5. Only values from proteins included in the protein signatures of this study are shown.

## Olink Proximity Extension Assay

Baseline serum samples from 273 PDAC patients were analyzed. Samples were randomized across Olink PEA (proximity extension assay) plates and normalized for any plate effects using the built-in inter-plate controls according to the manufacturer’s recommendations. The first five assay plates were analyzed prior to the remaining assay plates, and therefore eight patient samples from these first plates were included on all subsequent plates for bridging purposes.

Using a PEA, 1 µl of serum was mixed with pairs of antibodies linked to oligonucleotides (probes). Upon binding to the target antigen, the probes are brought into proximity with each other, leading to extension of the oligonucleotides by DNA polymerase. This acts as a surrogate marker for the specific antigen and can then be quantified by real-time PCR (qPCR), where the number of PCR copies are proportional to the initial concentration of antigen in the sample.^1, 2^

The PEA gives abundance levels for each protein measured as NPX values (Normalized Protein eXpression) on a log2 scale. Each assay has an experimentally determined lower limit of detection (LOD), defined as three standard deviations above the background level determined by the negative controls included on all plates. The standard deviations are assay specific and estimated during product validation for every panel. For assay values below LOD, the actual value was used. This was chosen because the LOD is considered a conservative measurement, and thus, there is a high probability that the value below the LOD is the real value. Also, this gives a less skewed distribution compared to replacing data below LOD with a value. Samples with minor quality control deviations were normalized and included in the analysis. Samples with major deviations were excluded from normalization and data analysis. Assay characteristics including detection limits calculations, assay performance, and validations are available from the manufacturer (www.olink.com).^2^

The analyses were performed at BioXpedia, Aarhus, Denmark, and were performed blinded to the study endpoints because no research questions were revealed before all samples had been analyzed.

| The REMARK Checklist^3^ | | **Page no.** |
| --- | --- | --- |
| **INTRODUCTION** | |  |
| 1 | State the marker examined, the study objectives, and any pre-specified hypotheses. | 8 |
| **MATERIALS AND METHODS** | |  |
| *Patients* | |  |
| 2 | Describe the characteristics (e.g., disease stage or co-morbidities) of the study patients, including their source and inclusion and exclusion criteria. | 9, Table 1 |
| 3 | Describe treatments received and how chosen (e.g., randomized or rule-based). | Table 1 |
| *Specimen characteristics* | |  |
| 4 | Describe type of biological material used (including control samples) and methods of preservation and storage. | 10 |
| *Assay methods* | |  |
| 5 | Specify the assay method used and provide (or reference) a detailed protocol, including specific reagents or kits used, quality control procedures, reproducibility assessments, quantitation methods, and scoring and reporting protocols. Specify whether and how assays were performed blinded to the study endpoint. | 10–11 |
| *Study design* | |  |
| 6 | State the method of case selection, including whether prospective or retrospective and whether stratification or matching (e.g., by stage of disease or age) was used. Specify the time period from which cases were taken, the end of the follow-up period, and the median follow-up time. | 9 |
| 7 | Precisely define all clinical endpoints examined. | 11–15 |
| 8 | List all candidate variables initially examined or considered for inclusion in models. | Table S1 |
| 9 | Give rationale for sample size; if the study was designed to detect a specified effect size, give the target power and effect size. | 9 |
| *Statistical analysis methods* | |  |
| 10 | Specify all statistical methods, including details of any variable selection procedures and other model-building issues, how model assumptions were verified, and how missing data were handled. | 11–15 |
| 11 | Clarify how marker values were handled in the analyses; if relevant, describe methods used for cutpoint determination. |  |
| **RESULTS** | |  |
| *Data* | |  |
| 12 | Describe the flow of patients through the study, including the number of patients included in each stage of the analysis (a diagram may be helpful) and reasons for dropout. Specifically, both overall and for each subgroup extensively examined report the numbers of patients and the number of events. | 16, figure S1 |
| 13 | Report distributions of basic demographic characteristics (at least age and sex), standard (disease-specific) prognostic variables, and tumor marker, including numbers of missing values. | Table 1 |
| *Analysis and presentation* | |  |
| 14 | Show the relation of the marker to standard prognostic variables. | 21–22 |
| 15 | Present univariable analyses showing the relation between the marker and outcome, with the estimated effect (e.g., hazard ratio and survival probability). Preferably provide similar analyses for all other variables being analyzed. For the effect of a tumor marker on a time-to-event outcome, a Kaplan-Meier plot is recommended. | Figure S2 |
| 16 | For key multivariable analyses, report estimated effects (e.g., hazard ratio) with confidence intervals for the marker and, at least for the final model, all other variables in the model. | *NA* |
| 17 | Among reported results, provide estimated effects with confidence intervals from an analysis in which the marker and standard prognostic variables are included, regardless of their statistical significance. | 16–19 |
| 18 | If done, report results of further investigations, such as checking assumptions, sensitivity analyses, and internal validation. | *NA* |
| **DISCUSSION** | |  |
| 19 | Interpret the results in the context of the pre-specified hypotheses and other relevant studies; include a discussion of limitations of the study. | 20–23 |
| 20 | Discuss implications for future research and clinical value. | 23 |

## TRIPOD guidelines checklist

| **Section/Topic** | **Item** | **Checklist Item** | **Page** |
| --- | --- | --- | --- |
| **Title and abstract** | | | |
| Title | 1 | Identify the study as developing and/or validating a multivariable prediction model, the target population, and the outcome to be predicted. | 1 |
| Abstract | 2 | Provide a summary of objectives, study design, setting, participants, sample size, predictors, outcome, statistical analysis, results, and conclusions. | 5 |
| **Introduction** | | | |
| Background and objectives | 3a | Explain the medical context (including whether diagnostic or prognostic) and rationale for developing or validating the multivariable prediction model, including references to existing models. | 6-7 |
|  | 3b | Specify the objectives, including whether the study describes the development or validation of the model or both. | 8 |
| **Methods** | | | |
| Source of data | 4a | Describe the study design or source of data (e.g., randomized trial, cohort, or registry data), separately for the development and validation data sets, if applicable. | 9 |
|  | 4b | Specify the key study dates, including start of accrual; end of accrual; and, if applicable, end of follow-up. | 9 |
| Participants | 5a | Specify key elements of the study setting (e.g., primary care, secondary care, general population) including number and location of centres. | 9 |
|  | 5b | Describe eligibility criteria for participants. | 9 |
|  | 5c | Give details of treatments received, if relevant. | 16 |
| Outcome | 6a | Clearly define the outcome that is predicted by the prediction model, including how and when assessed. | 11–14 |
|  | 6b | Report any actions to blind assessment of the outcome to be predicted. | *NA* |
| Predictors | 7a | Clearly define all predictors used in developing or validating the multivariable prediction model, including how and when they were measured. | 10, Table S1 |
|  | 7b | Report any actions to blind assessment of predictors for the outcome and other predictors. | 11 |
| Sample size | 8 | Explain how the study size was arrived at. | 9, 11 |
| Missing data | 9 | Describe how missing data were handled (e.g., complete-case analysis, single imputation, multiple imputation) with details of any imputation method. | *NA* |
| Statistical analysis methods | 10a | Describe how predictors were handled in the analyses. | 11–15 |
|  | 10b | Specify type of model, all model-building procedures (including any predictor selection), and method for internal validation. |  |
|  | 10d | Specify all measures used to assess model performance and, if relevant, to compare multiple models. |  |
| Risk groups | 11 | Provide details on how risk groups were created, if done. | *NA* |
| **Results** | | | |
| Participants | 13a | Describe the flow of participants through the study, including the number of participants with and without the outcome and, if applicable, a summary of the follow-up time. A diagram may be helpful. | Figure S1 |
|  | 13b | Describe the characteristics of the participants (basic demographics, clinical features, available predictors), including the number of participants with missing data for predictors and outcome. | Table 1 |
| Model development | 14a | Specify the number of participants and outcome events in each analysis. | 16–19 |
|  | 14b | If done, report the unadjusted association between each candidate predictor and outcome. | Table S2 |
| Model specification | 15a | Present the full prediction model to allow predictions for individuals (i.e., all regression coefficients, and model intercept or baseline survival at a given time point). | Upon request |
|  | 15b | Explain how to the use the prediction model. | *NA* |
| Model performance | 16 | Report performance measures (with CIs) for the prediction model. | 16–19 |
| **Discussion** | | | |
| Limitations | 18 | Discuss any limitations of the study (such as nonrepresentative sample, few events per predictor, missing data). | 23 |
| Interpretation | 19b | Give an overall interpretation of the results, considering objectives, limitations, and results from similar studies, and other relevant evidence. | 20–22 |
| Implications | 20 | Discuss the potential clinical use of the model and implications for future research. | 23 |
| **Other information** | | | |
| Supplementary information | 21 | Provide information about the availability of supplementary resources, such as study protocol, Web calculator, and data sets. | Supplement |
| Funding | 22 | Give the source of funding and the role of the funders for the present study. | 2 |

Abbreviations: *NA: not applicable*

# Supplementary Table S1: Full list of proteins in the immuno-oncology panel.

| **Abbreviated protein names** | **Protein names** | **UniProt ID** |
| --- | --- | --- |
| ADA | Adenosine deaminase | P00813 |
| ADGRG1 | Adhesion G-protein coupled receptor G1 | Q9Y653 |
| ANG-1 | Angiopoietin-1 | Q15389 |
| ANGPT2 | Angiopoietin-2 | O15123 |
| ARG1 | Arginase-1 | P05089 |
| CAIX | Carbonic anhydrase IX (CA9) | Q16790 |
| CASP-8 | Caspase-8 | Q14790 |
| CCL3 | C-C motif chemokine 3 | P10147 |
| CCL4 | C-C motif chemokine 4 | P13236 |
| CCL17 | C-C motif chemokine 17 | Q92583 |
| CCL19 | C-C motif chemokine 19 | Q99731 |
| CCL20 | C-C motif chemokine 20 | P78556 |
| CCL23 | C-C motif chemokine 23 | P55773 |
| CD4 | T-cell surface glycoprotein CD4 | P01730 |
| CD5 | T-cell surface glycoprotein CD5 | P06127 |
| CD8A | T-cell surface glycoprotein CD8 alpha chain | P01732 |
| CD27 | CD27 antigen | P26842 |
| CD28 | T-cell-specific surface glycoprotein CD28 | P10747 |
| CD40 | CD40L receptor | P25942 |
| CD40-L | CD40 ligand | P29965 |
| CD70 | CD70 antigen | P32970 |
| CD83 | CD83 antigen | Q01151 |
| CD244 | Natural killer cell receptor 2B4 | Q9BZW8 |
| CRTAM | Cytotoxic and regulatory T-cell molecule | O95727 |
| CSF-1 | Macrophage colony-stimulating factor 1 | P09603 |
| CX3CL1 | Fractalkine | P78423 |
| CXCL1 | C-X-C motif chemokine 1 | P09341 |
| CXCL5 | C-X-C motif chemokine 5 | P42830 |
| CXCL9 | C-X-C motif chemokine 9 | Q07325 |
| CXCL10 | C-X-C motif chemokine 10 | P02778 |
| CXCL11 | C-X-C motif chemokine 11 | O14625 |
| CXCL12 | Stromal cell-derived factor 1 | P48061 |
| CXCL13 | C-X-C motif chemokine 13 | O43927 |
| DCN | Decorin | P07585 |
| EGF | Pro-epidermal growth factor | P01133 |
| FASLG | Fas Ligand/Tumor necrosis factor ligand superfamily member 6 | P48023 |
| FGF2 | Fibroblast growth factor 2 | P09038 |
| Gal-1 | Galectin-1 | P09382 |
| Gal-9 | Galectin-9 | O00182 |
| GZMA | Granzyme A | P12544 |
| GZMB | Granzyme B | P10144 |
| GZMH | Granzyme H | P20718 |
| HGF | Hepatocyte growth factor | P14210 |
| HO-1 | Heme oxygenase 1 | P09601 |
| ICOSLG | ICOS ligand | O75144 |
| IFN-beta | Interferon beta | P01574 |
| IFN-gamma | Interferon gamma | P01579 |
| IL-1 alpha | Interleukin-1 alpha | P01583 |
| IL-2 | Interleukin-2 | P60568 |
| IL-4 | Interleukin-4 | P05112 |
| IL-5 | Interleukin-5 | P05113 |
| IL-6 | Interleukin-6 | P05231 |
| IL-7 | Interleukin-7 | P13232 |
| IL-8 | Interleukin 8 | P10145 |
| IL-10 | Interleukin-10 | P22301 |
| IL-12 | Interleukin-12 | P29459, P29460 |
| IL-12RB1 | Interleukin-12 receptor subunit beta-1 | P42701 |
| IL-13 | Interleukin-13 | P35225 |
| IL-18 | Interleukin-18 | Q14116 |
| IL-21 | Interleukin-21 | Q9HBE4 |
| IL-33 | Interleukin-33 | O95760 |
| IL-35 | Interleukin-35 | Q14213, P29459 |
| KLRD1 | Natural killer cells antigen CD94 | Q13241 |
| LAMP3 | Lysosome-associated membrane glycoprotein 3 | Q9UQV4 |
| LAP TGF-beta-1 | Latency-associated peptide transforming growth factor beta-1 | P01137 |
| MCP-1 | Monocyte chemotactic protein 1 | P13500 |
| MCP-2 | Monocyte chemotactic protein 2 | P80075 |
| MCP-3 | Monocyte chemotactic protein 3 | P80098 |
| MCP-4 | Monocyte chemotactic protein 4 | Q99616 |
| MIC-A/B | MHC class I polypeptide-related sequence A/B | Q29983, Q29980 |
| MMP7 | Matrix metalloproteinase-7 | P09237 |
| MMP12 | Macrophage metalloproteinase-12 | P39900 |
| NCR1 | Natural cytotoxicity triggering receptor | O76036 |
| NOS3 | Nitric oxide synthase, endothelia | P29474 |
| PDCD1 | Programmed cell death protein 1 | Q15116 |
| PDGF subunit B | Platelet-derived growth factor subunit B | P01127 |
| PD-L1 | Programmed cell death 1 ligand 1 | Q9NZQ7 |
| PD-L2 | Programmed cell death 1 ligand 2 | Q9BQ51 |
| PGF | Placenta growth factor | P49763 |
| PTN | Pleiotrophin | P21246 |
| TIE2 | Angiopoietin-1 receptor | Q02763 |
| TNF | Tumor necrosis factor | P01375 |
| TNFRSF4 | Tumor necrosis factor receptor superfamily member 4 | P43489 |
| TNFRSF9 | Tumor necrosis factor receptor superfamily member 9 | Q07011 |
| TNFRSF12A | Tumor necrosis factor receptor superfamily member 12A | Q9NP84 |
| TNFRSF21 | Tumor necrosis factor receptor superfamily member 21 | O75509 |
| TNFSF14 | Tumor necrosis factor ligand superfamily member 14 | O43557 |
| TRAIL | TNF-related apoptosis-inducing ligand | P50591 |
| TWEAK | Tumor necrosis factor ligand superfamily member 12 | O43508 |
| VEGFA | Vascular endothelial growth factor A | P15692 |
| VEGFC | Vascular endothelial growth factor C | P49767 |
| VEGFR-2 | Vascular endothelial growth factor receptor 2 | P35968 |

# Supplementary Table S2: Differential expressions of proteins between groups, only significant *P*-values given (unadjusted).

| **Protein** | **Comparison** | **LOD check** | **Test type** | ***P*-value** | **Adj. *P*-value** | **Median 1** | **Median 2** | **log2 FC** |
| --- | --- | --- | --- | --- | --- | --- | --- | --- |
| **CD244** | Resectable vs. Unresectable | Pass | *t-*test | **4.35e-03** | 6.59e-01 | 7.00e+00 | 7.10e+00 | -1.65e-01 |
| **CD5** | Resectable vs. Unresectable | Pass | *t-*test | **8.05e-03** | 6.59e-01 | 5.89e+00 | 6.08e+00 | -1.56e-01 |
| **ADA** | Resectable vs. Unresectable | Pass | *t-*test | **1.30e-02** | 6.59e-01 | 3.67e+00 | 3.91e+00 | -1.74e-01 |
| **VEGFC** | Resectable vs. Unresectable | Pass | *t-*test | **1.35e-02** | 6.59e-01 | 3.51e+00 | 3.62e+00 | -1.60e-01 |
| **MMP12** | Resectable vs. Unresectable | Pass | *t-*test | **1.88e-02** | 6.59e-01 | 6.78e+00 | 6.48e+00 | 2.45e-01 |
| **IL-18** | Resectable vs. Unresectable | Pass | *t-*test | **2.01e-02** | 6.59e-01 | 1.00e+01 | 1.02e+01 | -2.20e-01 |
| **IL-12** | Resectable vs. Unresectable | Pass | *t-*test | **2.05e-02** | 6.59e-01 | 7.68e+00 | 7.95e+00 | -2.59e-01 |
| **CCL17** | Resectable vs. Unresectable | Pass | *t-*test | **2.18e-02** | 6.59e-01 | 1.07e+01 | 1.03e+01 | 2.71e-01 |
| **IL-35** | Resectable vs. Unresectable | Flag | *t-*test | **2.40e-02** | 6.59e-01 | -4.05e-01 | -7.28e-01 | 1.99e-01 |
| **ICOSLG** | Resectable vs. Unresectable | Pass | *t-*test | **3.13e-02** | 6.59e-01 | 6.56e+00 | 6.64e+00 | -1.03e-01 |
| **CCL20** | Resectable vs. Unresectable | Pass | *t-*test | **3.63e-02** | 6.59e-01 | 8.56e+00 | 7.73e+00 | 4.84e-01 |
| **PDCD1** | Resectable vs. Unresectable | Pass | *t-*test | **3.65e-02** | 6.59e-01 | 4.14e+00 | 4.22e+00 | -1.73e-01 |
| **IFN-gamma** | Resectable vs. Unresectable | Pass | *t-*test | **4.29e-02** | 6.59e-01 | 1.05e+00 | 1.13e+00 | -7.15e-02 |
| **IL-35** | Resection: OS <median vs. OS >median | Flag | *t-test* | **9.76e-03** | 6.59e-01 | -6.78e-01 | -8.09e-01 | 2.65e-01 |
| **CD27** | Resection: OS <median vs. OS >median | Pass | *t-test* | **6.17e-03** | 6.59e-01 | 8.94e+00 | 9.16e+00 | -2.04e-01 |
| **PTN** | Resection: OS <median vs. OS >median | Pass | *t-test* | **7.35e-03** | 6.59e-01 | 2.95e+00 | 3.81e+00 | -6.72e-01 |
| **IFN-gamma** | Resection: OS <median vs. OS >median | Pass | *t-test* | **1.88e-02** | 6.59e-01 | 1.14e+00 | 1.11e+00 | 9.71e-02 |
| **CAIX** | Resection: OS <median vs. OS >median | Pass | *t-test* | **3.71e-02** | 6.59e-01 | 6.22e+00 | 6.36e+00 | -2.69e-01 |
| **IL-5** | Resection: OS <median vs. OS >median | Pass | *t-test* | **4.39e-02** | 6.59e-01 | 1.50e+00 | 1.03e+00 | 5.93e-01 |
| **CD27** | Resection: OS <1 year vs. OS >1 year | Pass | *t-*test | **1.37e-02** | 6.59e-01 | 8.94e+00 | 9.14e+00 | -1.70e-01 |
| **CCL4** | Resection: OS <1 year vs. OS >1 year | Pass | *t-*test | **2.46e-02** | 6.59e-01 | 9.46e+00 | 9.13e+00 | 2.28e-01 |
| **CD40-L** | Resection: OS <1 year vs. OS >1 year | Pass | *t-*test | **3.04e-02** | 6.59e-01 | 9.55e+00 | 9.38e+00 | 2.68e-01 |
| **GZMB** | Resection: OS <1 year vs. OS >1 year | Pass | *t-*test | **3.17e-02** | 6.59e-01 | 4.24e+00 | 3.87e+00 | 2.36e-01 |
| **CAIX** | Resection: OS <1 year vs. OS >1 year | Pass | *t-*test | **4.41e-02** | 6.59e-01 | 6.22e+00 | 6.31e+00 | -2.42e-01 |
| **FGF2** | Resection: OS <1 year vs. OS >1 year | Pass | *t-*test | **4.42e-02** | 6.59e-01 | 8.87e-01 | 7.24e-01 | 1.95e-01 |
| **IL-13** | Resection: OS <1 year vs. OS >3 years | Flag | Wilcoxon | **1.51e-02** | 6.59e-01 | 1.51e-02 | 5.53e-01 | -7.07e-01 |
| **CASP-8** | Resection: OS <1 year vs. OS >3 years | Pass | Wilcoxon | **2.67e-02** | 6.59e-01 | 6.62e+00 | 7.30e+00 | -7.25e-01 |
| **IL-12** | Resection: OS <1 year vs. OS >3 years | Pass | *t-*test | **4.56e-02** | 6.59e-01 | 7.65e+00 | 7.98e+00 | -2.89e-01 |
| **MCP-1** | Resection: OS <1 year vs. OS >4 years | Pass | Wilcoxon | **4.24e-03** | 6.59e-01 | 1.22e+01 | 1.24e+01 | -2.44e-01 |
| **CASP-8** | Resection: OS <1 year vs. OS >4 years | Pass | *t-*test | **6.08e-03** | 6.59e-01 | 6.83e+00 | 7.40e+00 | -4.77e-01 |
| **IL-8** | Resection: OS <1 year vs. OS >4 years | Pass | Wilcoxon | **1.34e-02** | 6.59e-01 | 8.13e+00 | 8.88e+00 | -3.83e-01 |
| **CAIX** | Resection: OS <1 year vs. OS >4 years | Pass | Wilcoxon | **1.71e-02** | 6.59e-01 | 5.86e+00 | 6.27e+00 | -3.50e-01 |
| **ARG1** | Resection: OS <1 year vs. OS >4 years | Pass | Wilcoxon | **3.48e-02** | 6.59e-01 | 3.90e+00 | 4.19e+00 | -3.59e-01 |
| **CD40** | Resection: OS <1 year vs. OS >4 years | Pass | Wilcoxon | **4.26e-02** | 6.59e-01 | 1.22e+01 | 1.23e+01 | -2.40e-01 |
| **MCP-1** | Resection: OS <1 year vs. OS >5 years | Pass | *t-*test | **9.25e-03** | 6.59e-01 | 1.22e+01 | 1.24e+01 | -2.14e-01 |
| **TNFRSF12A** | Resection: OS <1 year vs. OS >5 years | Pass | *t-*test | **2.56e-02** | 6.59e-01 | 7.08e+00 | 7.31e+00 | -2.59e-01 |
| **PTN** | Resection: OS <1 year vs. OS >5 years | Pass | *t-*test | **3.27e-02** | 6.59e-01 | 2.91e+00 | 3.44e+00 | -5.21e-01 |
| **IL-21** | Resection: OS <1 year vs. OS >5 years | Flag | *t-*test | **3.39e-02** | 6.59e-01 | 1.78e-01 | 2.93e-01 | -1.54e-01 |
| **MCP-2** | Resection: OS <1 year vs. OS >5 years | Pass | *t-*test | **4.18e-02** | 6.59e-01 | 8.73e+00 | 9.06e+00 | -2.71e-01 |
| **IFN-gamma** | Unresectable: OS <median vs. OS >median | Pass | Wilcoxon | **8.68e-03** | 6.59e-01 | 1.09e+00 | 9.86e-01 | 1.47e-01 |
| **CSF-1** | Unresectable: OS <median vs. OS >median | Pass | *t-*test | **1.76e-02** | 6.59e-01 | 9.20e+00 | 9.04e+00 | 1.62e-01 |
| **HGF** | Unresectable: OS <median vs. OS >median | Pass | *t-*test | **2.47e-02** | 6.59e-01 | 9.77e+00 | 9.65e+00 | 2.80e-01 |
| **TNFSF14** | Unresectable: OS <median vs. OS >median | Pass | *t-*test | **3.05e-02** | 6.59e-01 | 7.54e+00 | 7.26e+00 | 3.85e-01 |
| **PDGF-subunit-B** | Unresectable: OS <median vs. OS >median | Pass | Wilcoxon | **4.30e-02** | 6.59e-01 | 1.22e+01 | 1.22e+01 | 5.13e-02 |
| **IL-6** | Unresectable: OS <median vs. OS >median | Pass | Wilcoxon | **4.46e-02** | 6.59e-01 | 4.97e+00 | 4.63e+00 | 6.87e-01 |

Abbreviations: Wilcoxon = Wilcoxon rank sum test

# Supplementary Table S3: All tests run to determine the differential expressions of proteins between groups.

|  | **Resection**  **vs. non-resection** | | **Resection: OS <1 year vs. OS >1 year** | | **Resection: OS <1 year**  **vs. OS >3 years** | | **Resection: OS <1 year vs. OS >4 years** | | **Resection: OS <1 year**  **vs. OS >5 years** | | **No Resection: OS <median vs. OS >median** | | **Resection: OS <median vs. OS >median** | |
| --- | --- | --- | --- | --- | --- | --- | --- | --- | --- | --- | --- | --- | --- | --- |
| **Protein** | **Adj. *P*-value** | **Test type** | **Adj. *P*-value** | **Test type** | **Adj. *P*-value** | **Test type** | **Adj. *P*-value** | **Test type** | **Adj. *P*-value** | **Test type** | **Adj. *P*-value** | **Test type** | **Adj. *P*-value** | **Test type** |
| **ADA** | 6.59e-01 | *t-*test | 9.70e-01 | *t-*test | 7.18e-01 | Wilcoxon | 6.80e-01 | Wilcoxon | 7.20e-01 | *t-*test | 9.51e-01 | *t-*test | 7.65e-01 | *t-*test |
| **ADGRG1** | 1.00e+00 | *t-*test | 8.71e-01 | *t-*test | 6.98e-01 | Wilcoxon | 8.88e-01 | Wilcoxon | 9.51e-01 | *t-*test | 9.09e-01 | Wilcoxon | 8.43e-01 | *t-*test |
| **ANG-1** | 6.80e-01 | *t-*test | 7.38e-01 | *t-*test | 8.23e-01 | Wilcoxon | 7.20e-01 | Wilcoxon | 8.59e-01 | *t-*test | 7.38e-01 | Wilcoxon | 6.95e-01 | *t-*test |
| **ANGPT2** | 9.54e-01 | *t-*test | 9.95e-01 | *t-*test | 6.94e-01 | *t-*test | 1.00e+00 | *t-*test | 8.23e-01 | *t-*test | 6.80e-01 | *t-*test | 1.00e+00 | *t-*test |
| **ARG1** | 9.93e-01 | *t-*test | 1.00e+00 | *t-*test | 9.52e-01 | Wilcoxon | 6.59e-01 | Wilcoxon | 8.23e-01 | *t-*test | 6.80e-01 | *t-*test | 9.95e-01 | *t-*test |
| **CA19-9** | 7.38e-01 | *t-*test | 1.00e+00 | *t-*test | 9.74e-01 | Wilcoxon | 9.70e-01 | Wilcoxon | 1.00e+00 | *t-*test | 9.17e-01 | Wilcoxon | 9.56e-01 | *t-*test |
| **CAIX** | 9.74e-01 | *t-*test | 6.59e-01 | *t-*test | 6.94e-01 | Wilcoxon | 6.59e-01 | Wilcoxon | 7.38e-01 | *t-*test | 8.23e-01 | Wilcoxon | 6.59e-01 | *t-*test |
| **CASP-8** | 8.94e-01 | *t-*test | 9.70e-01 | *t-*test | 6.59e-01 | Wilcoxon | 6.59e-01 | *t-*test | 7.38e-01 | *t-*test | 7.81e-01 | Wilcoxon | 9.60e-01 | *t-*test |
| **CCL17** | 6.59e-01 | *t-*test | 9.63e-01 | *t-*test | 6.94e-01 | *t-*test | 9.95e-01 | *t-*test | 8.43e-01 | *t-*test | 9.95e-01 | *t-*test | 8.98e-01 | *t-*test |
| **CCL19** | 1.00e+00 | *t-*test | 9.77e-01 | *t-*test | 1.00e+00 | Wilcoxon | 8.25e-01 | Wilcoxon | 8.92e-01 | *t-*test | 9.70e-01 | *t-*test | 8.68e-01 | *t-*test |
| **CCL20** | 6.59e-01 | *t-*test | 8.88e-01 | *t-*test | 9.70e-01 | Wilcoxon | 8.73e-01 | Wilcoxon | 9.77e-01 | *t-*test | 9.92e-01 | Wilcoxon | 9.29e-01 | *t-*test |
| **CCL23** | 1.00e+00 | *t-*test | 9.84e-01 | *t-*test | 9.52e-01 | *t-*test | 9.95e-01 | *t-*test | 6.98e-01 | *t-*test | 6.80e-01 | *t-*test | 9.70e-01 | *t-*test |
| **CCL3** | 9.98e-01 | *t-*test | 8.68e-01 | *t-*test | 6.80e-01 | Wilcoxon | 7.38e-01 | Wilcoxon | 9.95e-01 | *t-*test | 8.50e-01 | *t-*test | 9.70e-01 | *t-*test |
| **CCL4** | 9.95e-01 | *t-*test | 6.59e-01 | *t-*test | 6.80e-01 | Wilcoxon | 9.70e-01 | Wilcoxon | 9.70e-01 | *t-*test | 9.70e-01 | *t-*test | 6.94e-01 | *t-*test |
| **CD244** | 6.59e-01 | *t-*test | 6.98e-01 | *t-*test | 7.38e-01 | Wilcoxon | 6.80e-01 | Wilcoxon | 1.00e+00 | *t-*test | 9.77e-01 | *t-*test | 8.92e-01 | *t-*test |
| **CD27** | 9.52e-01 | *t-*test | 6.59e-01 | *t-*test | 9.29e-01 | *t-*test | 9.70e-01 | *t-*test | 8.68e-01 | *t-*test | 8.63e-01 | Wilcoxon | 6.59e-01 | *t-*test |
| **CD28** | 8.73e-01 | *t-*test | 1.00e+00 | *t-*test | 7.75e-01 | Wilcoxon | 8.97e-01 | Wilcoxon | 9.54e-01 | *t-*test | 1.00e+00 | Wilcoxon | 9.54e-01 | *t-*test |
| **CD4** | 6.80e-01 | *t-*test | 8.98e-01 | *t-*test | 7.67e-01 | Wilcoxon | 8.98e-01 | Wilcoxon | 9.88e-01 | *t-*test | 9.56e-01 | Wilcoxon | 7.61e-01 | *t-*test |
| **CD40** | 9.95e-01 | *t-*test | 1.00e+00 | *t-*test | 6.80e-01 | Wilcoxon | 6.59e-01 | Wilcoxon | 6.94e-01 | *t-*test | 8.72e-01 | Wilcoxon | 9.51e-01 | *t-*test |
| **CD40-L** | 9.72e-01 | *t-*test | 6.59e-01 | *t-*test | 9.52e-01 | Wilcoxon | 8.98e-01 | Wilcoxon | 1.00e+00 | *t-*test | 8.75e-01 | Wilcoxon | 6.80e-01 | *t-*test |
| **CD5** | 6.59e-01 | *t-*test | 8.82e-01 | *t-*test | 8.23e-01 | *t-*test | 8.23e-01 | *t-*test | 9.12e-01 | *t-*test | 6.94e-01 | *t-*test | 6.98e-01 | *t-*test |
| **CD70** | 8.68e-01 | *t-*test | 9.93e-01 | *t-*test | 8.88e-01 | *t-*test | 8.36e-01 | *t-*test | 8.23e-01 | *t-*test | 8.73e-01 | *t-*test | 9.70e-01 | *t-*test |
| **CD83** | 6.80e-01 | *t-*test | 8.73e-01 | *t-*test | 7.38e-01 | *t-*test | 9.70e-01 | *t-*test | 8.23e-01 | *t-*test | 8.98e-01 | Wilcoxon | 7.36e-01 | *t-*test |
| **CD8A** | 8.63e-01 | *t-*test | 1.00e+00 | *t-*test | 1.00e+00 | Wilcoxon | 9.95e-01 | Wilcoxon | 9.56e-01 | *t-*test | 9.70e-01 | Wilcoxon | 8.23e-01 | *t-*test |
| **CRTAM** | 9.95e-01 | *t-*test | 8.73e-01 | *t-*test | 8.88e-01 | Wilcoxon | 9.77e-01 | Wilcoxon | 8.71e-01 | *t-*test | 9.74e-01 | *t-*test | 8.16e-01 | *t-*test |
| **CSF-1** | 9.74e-01 | *t-*test | 9.51e-01 | *t-*test | 6.98e-01 | Wilcoxon | 9.65e-01 | Wilcoxon | 8.92e-01 | *t-*test | 6.59e-01 | *t-*test | 8.81e-01 | *t-*test |
| **CX3CL1** | 9.85e-01 | *t-*test | 8.75e-01 | *t-*test | 9.65e-01 | *t-*test | 9.29e-01 | *t-*test | 9.52e-01 | *t-*test | 7.38e-01 | *t-*test | 7.20e-01 | *t-*test |
| **CXCL1** | 6.80e-01 | *t-*test | 8.73e-01 | *t-*test | 9.12e-01 | *t-*test | 9.95e-01 | *t-*test | 9.70e-01 | *t-*test | 6.80e-01 | *t-*test | 7.45e-01 | *t-*test |
| **CXCL10** | 6.80e-01 | *t-*test | 9.70e-01 | *t-*test | 9.54e-01 | Wilcoxon | 1.00e+00 | Wilcoxon | 9.12e-01 | *t-*test | 9.77e-01 | *t-*test | 8.73e-01 | *t-*test |
| **CXCL11** | 8.96e-01 | *t-*test | 1.00e+00 | *t-*test | 9.95e-01 | Wilcoxon | 9.70e-01 | *t-*test | 9.96e-01 | *t-*test | 9.95e-01 | *t-*test | 9.51e-01 | *t-*test |
| **CXCL12** | 9.92e-01 | *t-*test | 8.68e-01 | *t-*test | 9.95e-01 | Wilcoxon | 6.80e-01 | Wilcoxon | 6.94e-01 | *t-*test | 8.88e-01 | Wilcoxon | 9.52e-01 | *t-*test |
| **CXCL13** | 8.68e-01 | *t-*test | 9.77e-01 | *t-*test | 9.74e-01 | Wilcoxon | 6.94e-01 | Wilcoxon | 9.05e-01 | *t-*test | 8.88e-01 | *t-*test | 9.95e-01 | *t-*test |
| **CXCL5** | 8.88e-01 | *t-*test | 9.70e-01 | *t-*test | 6.80e-01 | Wilcoxon | 9.95e-01 | Wilcoxon | 9.05e-01 | *t-*test | 8.73e-01 | Wilcoxon | 9.12e-01 | *t-*test |
|  | **Resection**  **vs. non-resection** | | **Resection: OS <1 year vs. OS >1 year** | | **Resection: OS <1 year**  **vs. OS >3 years** | | **Resection: OS <1 year vs. OS >4 years** | | **Resection: OS <1 year**  **vs. OS >5 years** | | **No Resection: OS <median vs. OS >median** | | **Resection: OS <median vs. OS >median** | |
| **Protein** | **Adj. *P*-value** | **Test type** | **Adj. *P*-value** | **Test type** | **Adj. *P-*value** | **Test type** | **Adj. *P*-value** | **Test type** | **Adj. *P*-value** | **Test type** | **Adj. *P*-value** | **Test type** | **Adj. *P*-value** | **Test type** |
| **CXCL9** | 8.98e-01 | *t-*test | 8.23e-01 | *t-*test | 8.73e-01 | Wilcoxon | 7.38e-01 | Wilcoxon | 6.94e-01 | *t-*test | 8.68e-01 | *t-*test | 6.94e-01 | *t-*test |
| **DCN** | 6.80e-01 | *t-*test | 8.68e-01 | *t-*test | 9.95e-01 | Wilcoxon | 8.71e-01 | Wilcoxon | 8.20e-01 | *t-*test | 8.98e-01 | *t-*test | 7.20e-01 | *t-*test |
| **EGF** | 9.70e-01 | *t-*test | 6.80e-01 | *t-*test | 7.38e-01 | Wilcoxon | 8.65e-01 | Wilcoxon | 8.75e-01 | *t-*test | 6.98e-01 | Wilcoxon | 7.61e-01 | *t-*test |
| **FASLG** | 7.70e-01 | *t-*test | 9.26e-01 | *t-*test | 8.92e-01 | Wilcoxon | 9.70e-01 | Wilcoxon | 1.00e+00 | *t-*test | 8.73e-01 | *t-*test | 9.70e-01 | *t-*test |
| **FGF2** | 6.80e-01 | *t-*test | 6.59e-01 | *t-*test | 6.80e-01 | Wilcoxon | 6.98e-01 | Wilcoxon | 9.70e-01 | *t-*test | 6.80e-01 | Wilcoxon | 6.98e-01 | *t-*test |
| **Gal-1** | 8.23e-01 | *t-*test | 1.00e+00 | *t-*test | 8.73e-01 | *t-*test | 8.92e-01 | *t-*test | 8.73e-01 | *t-*test | 7.39e-01 | *t-*test | 7.38e-01 | *t-*test |
| **Gal-9** | 8.68e-01 | *t-*test | 8.88e-01 | *t-*test | 9.54e-01 | *t-*test | 9.63e-01 | *t-*test | 9.70e-01 | *t-*test | 8.88e-01 | *t-*test | 7.20e-01 | *t-*test |
| **GZMA** | 9.93e-01 | *t-*test | 8.23e-01 | *t-*test | 7.15e-01 | Wilcoxon | 8.98e-01 | Wilcoxon | 9.52e-01 | *t-*test | 9.52e-01 | *t-*test | 8.75e-01 | *t-*test |
| **GZMB** | 9.52e-01 | *t-*test | 6.59e-01 | *t-*test | 9.95e-01 | Wilcoxon | 9.72e-01 | Wilcoxon | 9.77e-01 | *t-*test | 9.29e-01 | Wilcoxon | 6.80e-01 | *t-*test |
| **GZMH** | 9.12e-01 | *t-*test | 8.37e-01 | *t-*test | 9.09e-01 | Wilcoxon | 9.12e-01 | Wilcoxon | 9.09e-01 | *t-*test | 7.20e-01 | Wilcoxon | 9.63e-01 | *t-*test |
| **HGF** | 9.93e-01 | *t-*test | 1.00e+00 | *t-*test | 8.73e-01 | Wilcoxon | 8.43e-01 | Wilcoxon | 9.52e-01 | *t-*test | 6.59e-01 | *t-*test | 9.93e-01 | *t-*test |
| **HO-1** | 9.29e-01 | *t-*test | 9.70e-01 | *t-*test | 8.72e-01 | Wilcoxon | 7.38e-01 | Wilcoxon | 1.00e+00 | *t-*test | 1.00e+00 | Wilcoxon | 9.70e-01 | *t-*test |
| **ICOSLG** | 6.59e-01 | *t-*test | 8.23e-01 | *t-*test | 8.43e-01 | Wilcoxon | 8.98e-01 | Wilcoxon | 9.70e-01 | *t-*test | 9.42e-01 | *t-*test | 6.80e-01 | *t-*test |
| **IFN-beta** | 9.83e-01 | *t-*test | 6.98e-01 | *t-*test | 8.73e-01 | Wilcoxon | 9.95e-01 | Wilcoxon | 8.98e-01 | *t-*test | 8.73e-01 | Wilcoxon | 8.23e-01 | *t-*test |
| **IFN-gamma** | 6.59e-01 | *t-*test | 6.80e-01 | *t-*test | 9.77e-01 | Wilcoxon | 7.38e-01 | Wilcoxon | 6.80e-01 | *t-*test | 6.59e-01 | Wilcoxon | 6.59e-01 | *t-*test |
| **IL-1-alpha** | 9.51e-01 | *t-*test | 6.80e-01 | *t-*test | 9.95e-01 | Wilcoxon | 9.95e-01 | Wilcoxon | 9.85e-01 | *t-*test | 9.77e-01 | Wilcoxon | 7.20e-01 | *t-*test |
| **IL-2** | 6.94e-01 | *t-*test | 1.00e+00 | *t-*test | 6.80e-01 | Wilcoxon | 1.00e+00 | Wilcoxon | 8.43e-01 | *t-*test | 9.70e-01 | *t-*test | 9.95e-01 | *t-*test |
| **IL-4** | 8.92e-01 | *t-*test | 6.80e-01 | *t-*test | 9.51e-01 | Wilcoxon | 1.00e+00 | Wilcoxon | 1.00e+00 | *t-*test | 9.05e-01 | Wilcoxon | 6.80e-01 | *t-*test |
| **IL-5** | 9.87e-01 | *t-*test | 7.05e-01 | *t-*test | 8.81e-01 | Wilcoxon | 6.80e-01 | Wilcoxon | 7.15e-01 | *t-*test | 9.95e-01 | Wilcoxon | 6.59e-01 | *t-*test |
| **IL-6** | 6.80e-01 | *t-*test | 9.74e-01 | *t-*test | 8.98e-01 | Wilcoxon | 9.29e-01 | Wilcoxon | 9.95e-01 | *t-*test | 6.59e-01 | Wilcoxon | 9.70e-01 | *t-*test |
| **IL-7** | 8.73e-01 | *t-*test | 9.70e-01 | *t-*test | 9.77e-01 | *t-*test | 9.95e-01 | *t-*test | 9.88e-01 | *t-*test | 8.88e-01 | *t-*test | 9.95e-01 | *t-*test |
| **IL-8** | 6.98e-01 | *t-*test | 8.73e-01 | *t-*test | 6.80e-01 | Wilcoxon | 6.59e-01 | Wilcoxon | 8.92e-01 | *t-*test | 6.80e-01 | *t-*test | 8.73e-01 | *t-*test |
| **IL-10** | 8.98e-01 | *t-*test | 7.20e-01 | *t-*test | 8.43e-01 | Wilcoxon | 9.66e-01 | Wilcoxon | 8.23e-01 | *t-*test | 6.80e-01 | Wilcoxon | 6.96e-01 | *t-*test |
| **IL-12** | 6.59e-01 | *t-*test | 1.00e+00 | *t-*test | 6.59e-01 | *t-*test | 9.12e-01 | *t-*test | 9.88e-01 | *t-*test | 9.93e-01 | *t-*test | 8.88e-01 | *t-*test |
| **IL-12RB1** | 7.45e-01 | *t-*test | 9.95e-01 | *t-*test | 8.92e-01 | Wilcoxon | 9.93e-01 | Wilcoxon | 9.74e-01 | *t-*test | 9.70e-01 | *t-*test | 8.88e-01 | *t-*test |
| **IL-13** | 9.70e-01 | *t-*test | 8.44e-01 | *t-*test | 6.59e-01 | Wilcoxon | 6.80e-01 | Wilcoxon | 6.94e-01 | *t-*test | 8.96e-01 | Wilcoxon | 6.80e-01 | *t-*test |
| **IL-18** | 6.59e-01 | *t-*test | 8.23e-01 | *t-*test | 9.51e-01 | *t-*test | 9.70e-01 | Wilcoxon | 9.95e-01 | *t-*test | 8.71e-01 | *t-*test | 9.52e-01 | *t-*test |
| **IL-21** | 7.38e-01 | *t-*test | 9.70e-01 | *t-*test | 6.80e-01 | Wilcoxon | 6.94e-01 | Wilcoxon | 6.59e-01 | *t-*test | 8.75e-01 | Wilcoxon | 1.00e+00 | *t-*test |
| **IL-33** | 9.54e-01 | *t-*test | 6.94e-01 | *t-*test | 9.54e-01 | Wilcoxon | 7.38e-01 | Wilcoxon | 8.23e-01 | *t-*test | 6.80e-01 | *t-*test | 6.80e-01 | *t-*test |
| **IL-35** | 6.59e-01 | *t-*test | 6.80e-01 | *t-*test | 9.12e-01 | Wilcoxon | 9.51e-01 | Wilcoxon | 9.95e-01 | *t-*test | 8.75e-01 | Wilcoxon | 6.59e-01 | *t-*test |
| **KLRD1** | 8.92e-01 | *t-*test | 9.95e-01 | *t-*test | 8.23e-01 | *t-*test | 6.94e-01 | Wilcoxon | 1.00e+00 | *t-*test | 9.95e-01 | *t-*test | 9.95e-01 | *t-*test |
| **LAMP3** | 9.93e-01 | *t-*test | 9.95e-01 | *t-*test | 9.70e-01 | Wilcoxon | 8.23e-01 | Wilcoxon | 9.95e-01 | *t-*test | 8.73e-01 | *t-*test | 9.54e-01 | *t-*test |
| **LAP-TGF-beta-1** | 9.70e-01 | *t-*test | 9.77e-01 | *t-*test | 7.80e-01 | Wilcoxon | 8.98e-01 | *t-*test | 9.93e-01 | *t-*test | 9.05e-01 | *t-*test | 1.00e+00 | *t-*test |
| **MCP-1** | 7.20e-01 | *t-*test | 9.77e-01 | *t-*test | 7.20e-01 | Wilcoxon | 6.59e-01 | Wilcoxon | 6.59e-01 | *t-*test | 7.38e-01 | *t-*test | 9.09e-01 | *t-*test |
| **MCP-2** | 8.23e-01 | *t-*test | 9.98e-01 | *t-*test | 8.23e-01 | Wilcoxon | 6.94e-01 | Wilcoxon | 6.59e-01 | *t-*test | 8.72e-01 | *t-*test | 1.00e+00 | *t-*test |
| **MCP-3** | 8.88e-01 | *t-*test | 9.71e-01 | *t-*test | 7.65e-01 | Wilcoxon | 7.39e-01 | Wilcoxon | 8.73e-01 | *t-*test | 9.29e-01 | Wilcoxon | 9.95e-01 | *t-*test |
|  | **Resection**  **vs. non-resection** | | **Resection: OS <1 year vs. OS >1 year** | | **Resection: OS <1 year**  **vs. OS >3 years** | | **Resection: OS <1 year vs. OS >4 years** | | **Resection: OS <1 year**  **vs. OS >5 years** | | **No Resection: OS <median vs. OS >median** | | **Resection: OS <median vs. OS >median** | |
| **Protein** | **Adj. *P*-value** | **Test type** | **Adj. *P*-value** | **Test type** | **Adj. *P*-value** | **Test type** | **Adj. *P*-value** | **Test type** | **Adj. *P-*value** | **Test type** | **Adj. *P*-value** | **Test type** | **Adj. *P*-value** | **Test type** |
| **MCP-4** | 8.43e-01 | *t-*test | 9.96e-01 | *t-*test | 9.51e-01 | *t-*test | 6.80e-01 | *t-*test | 7.80e-01 | *t-*test | 7.45e-01 | *t-*test | 1.00e+00 | *t-*test |
| **MIC-A-B** | 6.80e-01 | *t-*test | 9.70e-01 | *t-*test | 7.20e-01 | Wilcoxon | 8.66e-01 | Wilcoxon | 9.70e-01 | *t-*test | 9.77e-01 | Wilcoxon | 8.98e-01 | *t-*test |
| **MMP12** | 6.59e-01 | *t-*test | 9.12e-01 | *t-*test | 6.80e-01 | *t-*test | 8.98e-01 | *t-*test | 9.53e-01 | *t-*test | 9.51e-01 | *t-*test | 9.65e-01 | *t-*test |
| **MMP7** | 8.73e-01 | *t-*test | 9.12e-01 | *t-*test | 6.80e-01 | Wilcoxon | 7.94e-01 | Wilcoxon | 6.80e-01 | *t-*test | 6.94e-01 | Wilcoxon | 9.54e-01 | *t-*test |
| **NCR1** | 6.80e-01 | *t-*test | 9.52e-01 | *t-*test | 8.92e-01 | *t-*test | 8.71e-01 | *t-*test | 6.80e-01 | *t-*test | 8.98e-01 | *t-*test | 9.54e-01 | *t-*test |
| **NOS3** | 9.96e-01 | *t-*test | 7.38e-01 | *t-*test | 9.05e-01 | Wilcoxon | 7.38e-01 | Wilcoxon | 9.95e-01 | *t-*test | 8.88e-01 | Wilcoxon | 7.20e-01 | *t-*test |
| **PDCD1** | 6.59e-01 | *t-*test | 9.70e-01 | *t-*test | 8.68e-01 | Wilcoxon | 1.00e+00 | Wilcoxon | 1.00e+00 | *t-*test | 9.70e-01 | *t-*test | 8.63e-01 | *t-*test |
| **PDGF-subunit-B** | 6.80e-01 | *t-*test | 9.75e-01 | *t-*test | 8.88e-01 | Wilcoxon | 9.54e-01 | *t-*test | 9.88e-01 | *t-*test | 6.59e-01 | Wilcoxon | 9.52e-01 | *t-*test |
| **PD-L1** | 6.80e-01 | *t-*test | 9.51e-01 | *t-*test | 8.68e-01 | Wilcoxon | 1.00e+00 | Wilcoxon | 8.72e-01 | *t-*test | 7.47e-01 | Wilcoxon | 9.77e-01 | *t-*test |
| **PD-L2** | 9.72e-01 | *t-*test | 9.52e-01 | *t-*test | 8.68e-01 | Wilcoxon | 9.12e-01 | Wilcoxon | 1.00e+00 | *t-*test | 9.93e-01 | *t-*test | 9.70e-01 | *t-*test |
| **PGF** | 9.77e-01 | *t-*test | 8.73e-01 | *t-*test | 8.73e-01 | Wilcoxon | 8.68e-01 | Wilcoxon | 9.29e-01 | *t-*test | 7.80e-01 | Wilcoxon | 8.43e-01 | *t-*test |
| **PTN** | 9.95e-01 | *t-*test | 6.80e-01 | *t-*test | 8.24e-01 | Wilcoxon | 7.38e-01 | Wilcoxon | 6.59e-01 | *t-*test | 8.92e-01 | *t-*test | 6.59e-01 | *t-*test |
| **TIE2** | 9.96e-01 | *t-*test | 8.43e-01 | *t-*test | 9.70e-01 | *t-*test | 8.88e-01 | *t-*test | 6.80e-01 | *t-*test | 9.74e-01 | Wilcoxon | 7.38e-01 | *t-*test |
| **TNF** | 8.43e-01 | *t-*test | 9.51e-01 | *t-*test | 9.54e-01 | Wilcoxon | 1.00e+00 | Wilcoxon | 9.80e-01 | *t-*test | 8.43e-01 | Wilcoxon | 9.88e-01 | *t-*test |
| **TNFRSF12A** | 9.74e-01 | *t-*test | 6.80e-01 | *t-*test | 6.98e-01 | Wilcoxon | 6.80e-01 | Wilcoxon | 6.59e-01 | *t-*test | 8.23e-01 | Wilcoxon | 6.80e-01 | *t-*test |
| **TNFRSF21** | 8.23e-01 | *t-*test | 9.09e-01 | *t-*test | 8.23e-01 | *t-*test | 9.46e-01 | *t-*test | 8.23e-01 | *t-*test | 8.20e-01 | *t-*test | 8.05e-01 | *t-*test |
| **TNFRSF4** | 7.38e-01 | *t-*test | 8.73e-01 | *t-*test | 9.60e-01 | Wilcoxon | 7.20e-01 | Wilcoxon | 9.52e-01 | *t-*test | 8.71e-01 | Wilcoxon | 7.20e-01 | *t-*test |
| **TNFRSF9** | 9.52e-01 | *t-*test | 7.19e-01 | *t-*test | 6.80e-01 | Wilcoxon | 9.52e-01 | Wilcoxon | 6.80e-01 | *t-*test | 8.75e-01 | *t-*test | 6.80e-01 | *t-*test |
| **TNFSF14** | 8.92e-01 | *t-*test | 6.80e-01 | *t-*test | 6.80e-01 | *t-*test | 6.80e-01 | *t-*test | 9.93e-01 | *t-*test | 6.59e-01 | *t-*test | 8.23e-01 | *t-*test |
| **TRAIL** | 6.80e-01 | *t-*test | 9.52e-01 | *t-*test | 6.80e-01 | Wilcoxon | 9.54e-01 | *t-*test | 8.88e-01 | *t-*test | 1.00e+00 | Wilcoxon | 8.92e-01 | *t-*test |
| **TWEAK** | 9.12e-01 | *t-*test | 6.94e-01 | *t-*test | 8.37e-01 | *t-*test | 9.95e-01 | *t-*test | 9.70e-01 | *t-*test | 9.56e-01 | Wilcoxon | 8.72e-01 | *t-*test |
| **VEGFA** | 8.79e-01 | *t-*test | 8.98e-01 | *t-*test | 9.95e-01 | *t-*test | 1.00e+00 | *t-*test | 9.74e-01 | *t-*test | 8.43e-01 | *t-*test | 9.77e-01 | *t-*test |
| **VEGFC** | 6.59e-01 | *t-*test | 7.81e-01 | *t-*test | 8.68e-01 | Wilcoxon | 8.09e-01 | Wilcoxon | 9.87e-01 | *t-*test | 8.16e-01 | *t-*test | 8.92e-01 | *t-*test |
| **VEGFR-2** | 1.00 | *t-*test | 8.23e-01 | *t-*test | 9.70e-01 | *t-*test | 9.95e-01 | *t-*test | 8.73e-01 | *t-*test | 6.80e-01 | *t-*test | 8.43e-01 | *t-*test |

*P*-values marked in red are flagged because the values from which the *P*-values have been calculated from are lower than the limit of detection for the assay.

Abbreviations: Wilcoxon = Wilcoxon rank sum test.

# Supplementary Table S4: Performance of the prognostic plasma protein signatures from the comparisons: Resectable with OS <1 year vs. >3 years (A1–6), Resectable with OS <median vs. >median (OS 22.6 months; B1–5), and Unresectable with OS <median vs. >median (OS 8.2 months; C1–5).

|  | **Signature** | **AUC (95% CI)** | **BPsens (95% CI)** | **BPspec (95% CI)** | **PPV**  **(95% CI)** | **NPV**  **(95% CI)** | **AUC (95% CI)** | **BPsens (95% CI)** | **BPspec (95% CI)** | **PPV**  **(95% CI)** | **NPV (95% CI)** |
| --- | --- | --- | --- | --- | --- | --- | --- | --- | --- | --- | --- |
| **Resectable with OS <median vs. >median** | **A1** | **Discovery cohort (n = 123)** | | | | | **Replication cohort (n = 70)** | | | | |
|  |  | 0.52 (0.37–0.68) | 0.14 (0.09–0.95) | 1.0 (0.21–1.0) | 1.0 (0.72–1.0) | 0.34 (0.33–0.75) | 0.55 (0.38–0.73) | 0.60 (0.12–1.0) | 0.66 (0.16–1.0) | 0.89 (0.85–1.0) | 0.25 (0.18–1.0) |
|  | **A2** | 0.74 (0.60–0.88) | 0.78 (0.42–1.0) | 0.68 (0.36–0.94) | 0.84 (0.77–0.96) | 0.59 (0.42–1.0) | 0.62 (0.45–0.80) | 0.58 (0.17–0.96) | 0.75 (0.33–1.0) | 0.91 (0.86–1.0) | 0.27 (0.20–0.66) |
|  | **A3** | 0.75 (0.60–0.9) | 0.83 (0.59–1.0) | 0.68 (0.36–0.89) | 0.85 (0.77–0.94) | 0.65 (0.49–1.0) | 0.62 (0.46–0.79) | 0.43 (0.24–1.0) | 0.83 (0.25–1.0) | 0.92 (0.86–1.0) | 0.23 (0.20–1.0) |
|  | **A4** | 0.71 (0.55–0.86) | 0.73 (0.52–0.97) | 0.68 (0.36–0.94) | 0.83 (0.76–0.95) | 0.54 (0.42–0.90) | 0.48 (0.30–0.66) | 0.50 (0.01–0.94) | 0.58 (0.16–1.0) | 0.85 (0.83–1.0) | 0.19 (0.17–0.40) |
|  | **A5** | 0.69 (0.53–0.84) | 0.64 (0.52–0.90) | 0.78 (0.47–0.94) | 0.87 (0.76–0.96) | 0.50 (0.41–0.76) | 0.52 (0.34–0.70) | 0.32 (0.10–0.91) | 0.83 (0.25–1.0) | 0.9 (0.84–1.0) | 0.20 (0.17–0.42) |

| **Resectable with OS <1 year vs. >3 years** | **B1** | **Discovery cohort (n = 74)** | | | | | **Replication cohort (n = 50)** | | | | |
| --- | --- | --- | --- | --- | --- | --- | --- | --- | --- | --- | --- |
|  |  | 0.65 (0.45–0.84) | 0.55 (0.33–0.77) | 0.94 (0.78–1.0) | 0.90 (0.71–1.0) | 0.69 (0.59–0.82) | 0.58 (0.42–0.74) | 0.77 (0.18–0.96) | 0.43 (0.21–1.0) | 0.61 (0.57–1.0) | 0.62 (0.50–0.87) |
|  | **B2** | 0.77 (0.60–0.94) | 0.83 (0.61–1.0) | 0.78 (0.63–1.0) | 0.78 (0.68–1.0) | 0.83 (0.69–1.0) | 0.62 (0.47–0.78) | 0.51 (0.29–0.85) | 0.82 (0.43–1.0) | 0.77 (0.63–1.0) | 0.59 (0.51–0.76) |
|  | **B3** | 0.74 (0.57–0.91) | 0.72 (0.50–0.94) | 0.84 (0.68–1.0) | 0.81 (0.66–1.0) | 0.76 (0.65–0.93) | 0.63 (0.47–0.79) | 0.55 (0.18–0.88) | 0.73 (0.39–1.0) | 0.71 (0.60–1.0) | 0.58 (0.51–0.83) |
|  | **B4** | 0.72 (0.55–0.90) | 0.66 (0.44–0.94) | 0.78 (0.52–1.0) | 0.75 (0.61–1.0) | 0.71 (0.62–0.93) | 0.61 (0.45–0.77) | 0.59 (0.18–0.92) | 0.69 (0.34–1.0) | 0.69 (0.60–1.0) | 0.59 (0.50–0.83) |
|  | **B5** | 0.69 (0.50–0.87) | 0.61 (0.38–0.94) | 0.84 (0.52–1.0) | 0.78 (0.61–1.0) | 0.69 (0.59–0.89) | 0.62 (0.46–0.79) | 0.81 (0.55–1.0) | 0.52 (0.26–0.86) | 0.66 (0.59–0.84) | 0.7 (0.56–1.0) |
|  | **B6** | 0.65 (0.45–0.84) | 0.66 (0.27–0.88) | 0.68 (0.52–1.0) | 0.66 (0.59–1.0) | 0.68 (0.58–0.87) | 0.64 (0.48–0.80) | 0.66 (0.48–1.0) | 0.65 (0.26–0.86) | 0.69 (0.59–0.85) | 0.62 (0.54–1.0) |
| **Unresectable with OS <median vs. >median** | **C1** | **Discovery cohort (n = 51)** | | | | | **Replication cohort (n = 29)** | | | | |
|  |  | 0.54 (0.30–0.77) | 0.60 (0.13–1.0) | 0.60 (0.20–1.0) | 0.69 (0.63–1.0) | 0.50 (0.43–1.0) | 0.47 (0.22–0.72) | 0.95 (0.10–1.0) | 0.22 (0.11–1.0) | 0.73 (0.69–1.0) | 0.66 (0.32–1.0) |
|  | **C2** | 0.78 (0.58–0.99) | 0.86 (0.53–1.0) | 0.70 (0.50–1.0) | 0.81 (0.73–1.0) | 0.77 (0.56–1.0) | 0.62 (0.40–0.85) | 0.60 (0.30–1.0) | 0.77 (0.22–1.0) | 0.85 (0.73–1.0) | 0.46 (0.36–1.0) |
|  | **C3** | 0.78 (0.56–1.0) | 1.0 (0.66–1.0) | 0.60 (0.40–1.0) | 0.78 (0.71–1.0) | 1.0 (0.63–1.0) | 0.64 (0.42–0.86) | 0.65 (0.25–1.0) | 0.77 (0.33–1.0) | 0.86 (0.75–1.0) | 0.50 (0.36–1.0) |
|  | **C4** | 0.68 (0.45–0.92) | 0.93 (0.33–1.0) | 0.50 (0.30–1.0) | 0.73 (0.66–1.0) | 0.83 (0.49–1.0) | 0.63 (0.39–0.88) | 0.80 (0.35–1.0) | 0.55 (0.22–1.0) | 0.80 (0.73–1.0) | 0.55 (0.36–1.0) |
|  | **C5** | 0.74 (0.52–0.95) | 0.93 (0.40–1.0) | 0.60 (0.40–1.0) | 0.77 (0.68–1.0) | 0.85 (0.50–1.0) | 0.55 (0.32–0.79) | 0.75 (0.10–0.95) | 0.44 (0.33–1.0) | 0.75 (0.72–1.0) | 0.44 (0.33–0.83) |

# Supplementary Table S5: Mean NPX values of proteins included in all our protein signatures compared to NPX values of the misclassified patient.

| **Protein** | **Mean NPX values** | | **One patient misclassified as resectable** |
| --- | --- | --- | --- |
|  | **Resectable** | **Unresectable** |  |
| **ADA** | 3.93676 | 3.762702 | 3.03519 |
| **ADGRG1** | 3.191424 | 3.186837 | 1.926605 |
| **CAIX** | 6.322068 | 6.356351 | 5.33291 |
| **CCL20** | 7.95857171 | 8.442226 | 4.383605 |
| **CD5** | 6.078983575 | 5.922806 | 5.05046 |
| **CD8A** | 10.60626422 | 10.45965 | 9.85655 |
| **CD27** | 9.018971477 | 8.989004 | 8.142775 |
| **CD40** | 12.40239368 | 12.41269 | 11.58209 |
| **CD40L** | 9.441615 | 9.411248 | 9.313605 |
| **CD244** | 7.129276528 | 6.96407 | 6.295725 |
| **CXCL1** | 11.05527347 | 11.18624 | 9.97307 |
| **CXCL9** | 9.214035155 | 9.133923 | 8.24265 |
| **FGF2** | 0.967897047 | 1.193347 | 1.096255 |
| **GZMB** | 4.167959974 | 4.123742 | 3.995 |
| **GZMH** | 6.028994741 | 6.10491 | 5.41088 |
| **IFN–gamma** | 1.163660674 | 1.092125 | 0.906505 |
| **IL–1 alpha** | –0.878896166 | –0.92725 | –1.01946 |
| **IL–4** | 0.081413886 | –0.004868063 | 0.47392 |
| **IL–5** | 1.919406788 | 1.867008 | 9.561795 |
| **IL–6** | 4.785448005 | 5.109232 | 3.455135 |
| **IL–10** | 4.545920881 | 4.63146 | 3.824765 |
| **IL–12** | 8.007007979 | 7.748062 | 6.29822 |
| **IL–33** | 1.047585 | 0.642543 | 0.656298523 |
| **MIC–A/B** | 5.806892358 | 5.354452 | 5.849245 |
| **PTN** | 3.295228238 | 3.272259 | 1.558735 |
| **VEGF–C** | 3.641234067 | 3.481318 | 2.86775 |
| **VEGFR–2** | 7.847863212 | 7.848693 | 7.577175 |
| **CA19–9** | 7.315777586 | 7.872819 | 11.45121111 |

# Supplementary Figure S1: Patient–flow for the two statistical approaches

a) Flow according to statistical approach


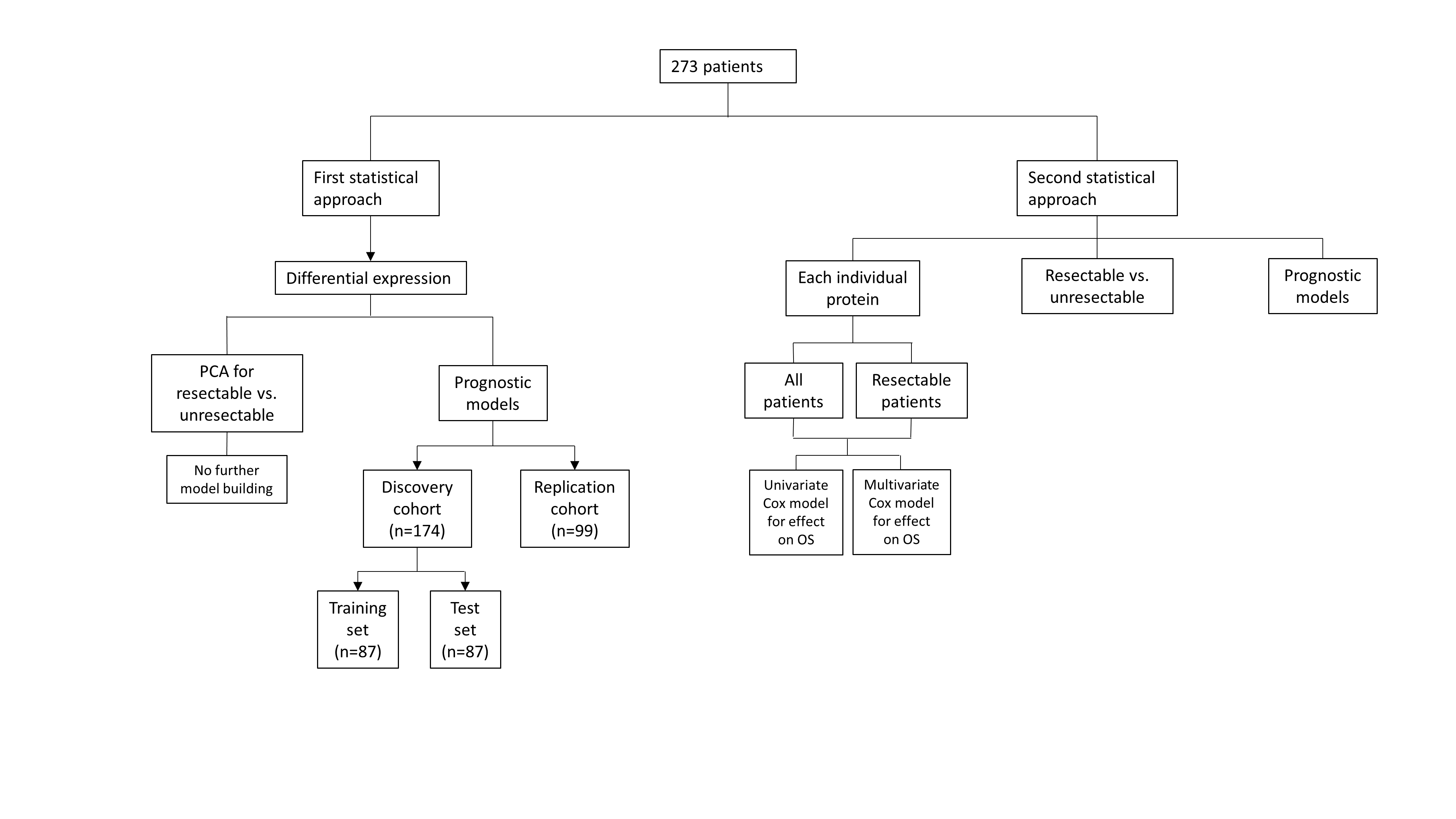


b) Flow according to results


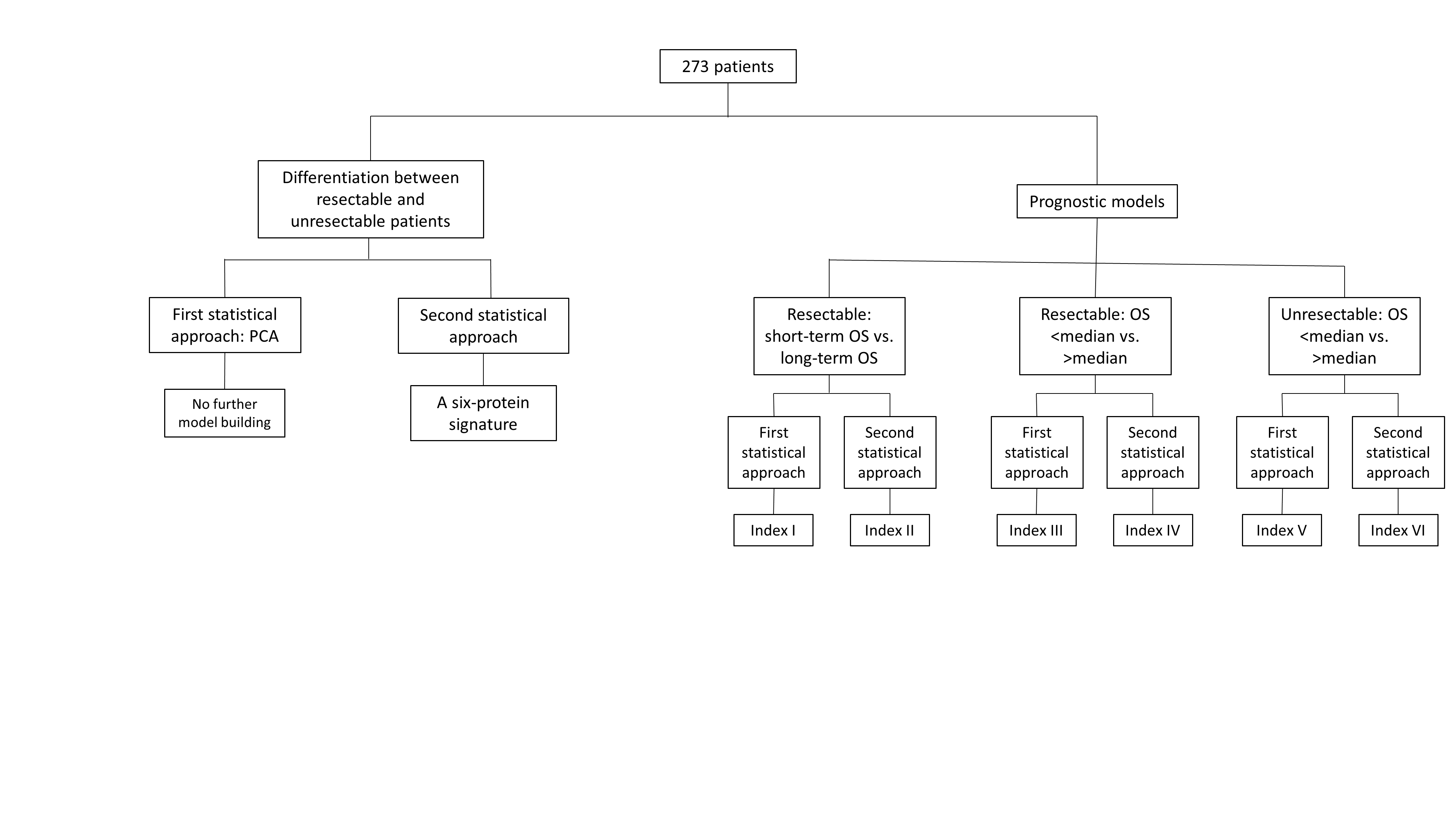


# Supplementary Figure S2: Kaplan-Meier curves of proteins with *P* < 0.05. Divided according to NPX values (< median or > median).

## A) All patients, n = 273


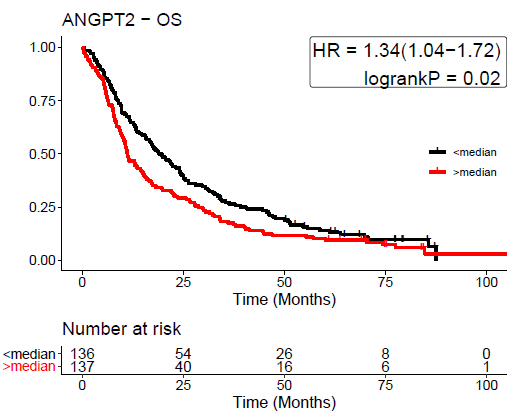

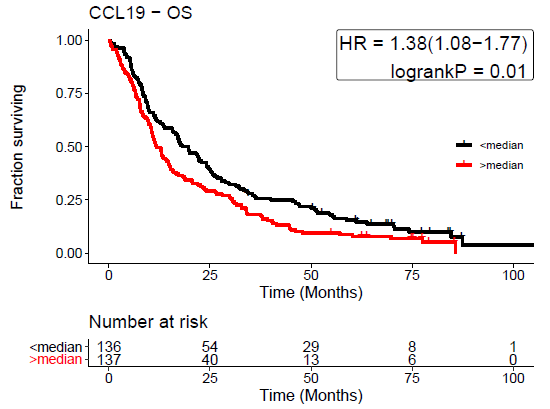


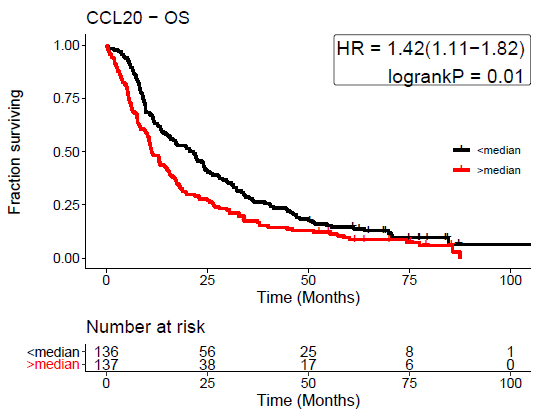

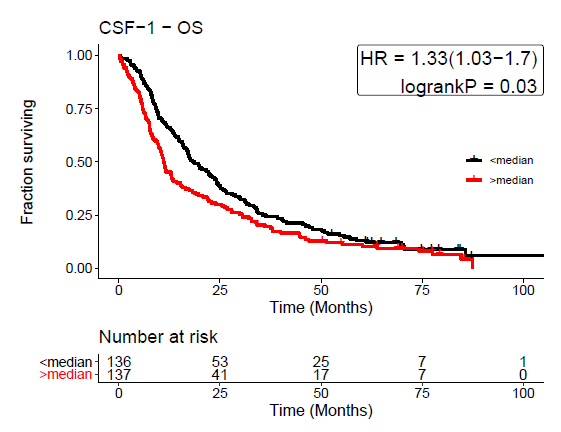


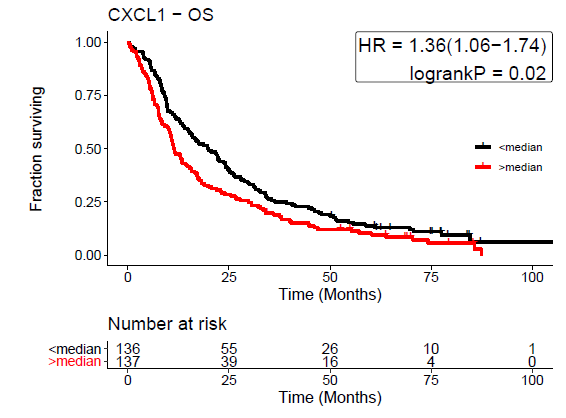

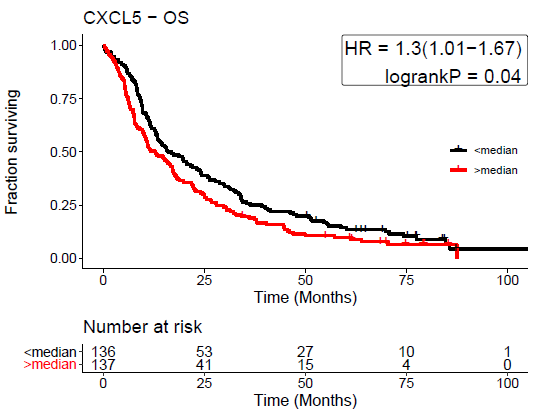


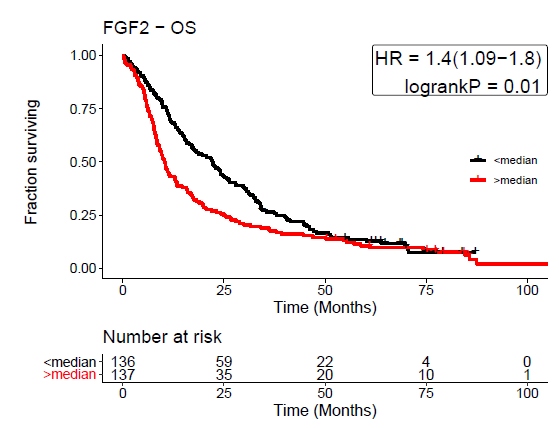

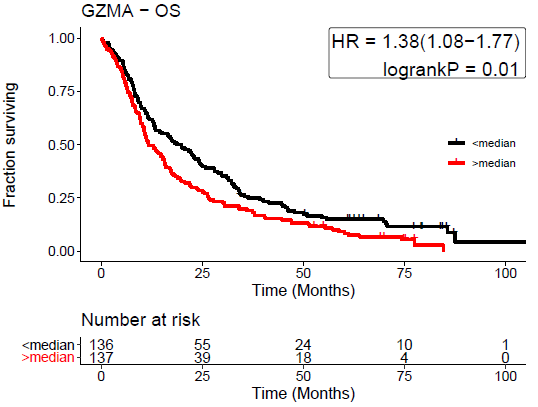


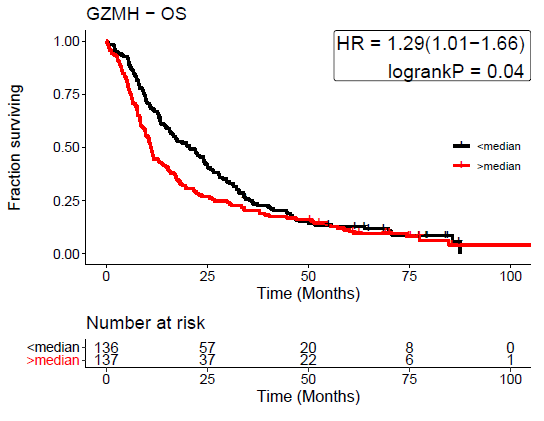

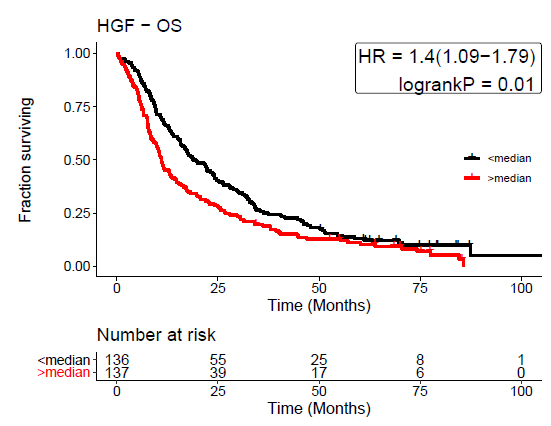


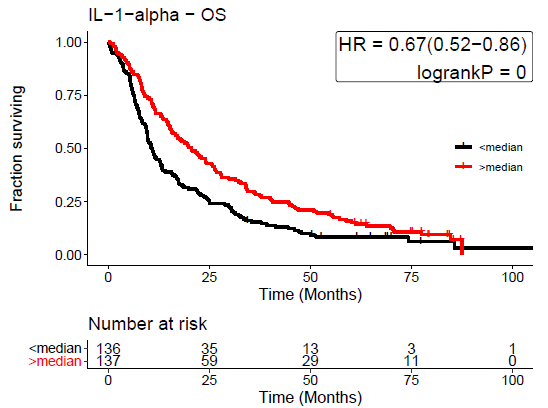

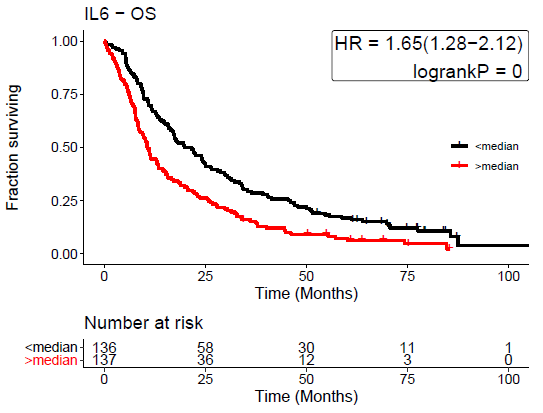


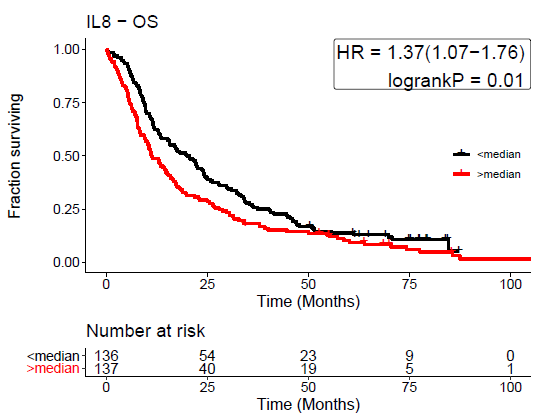

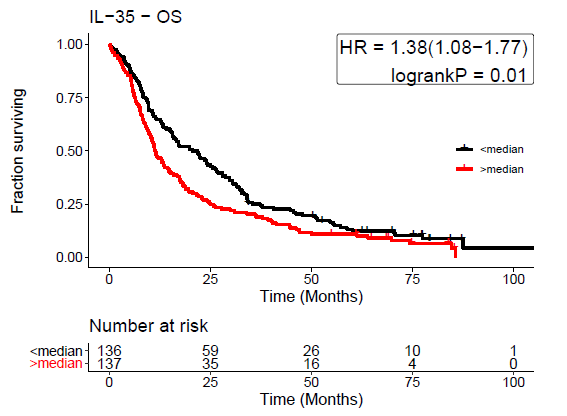


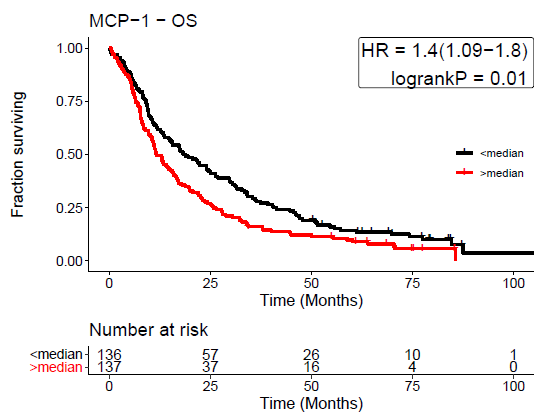

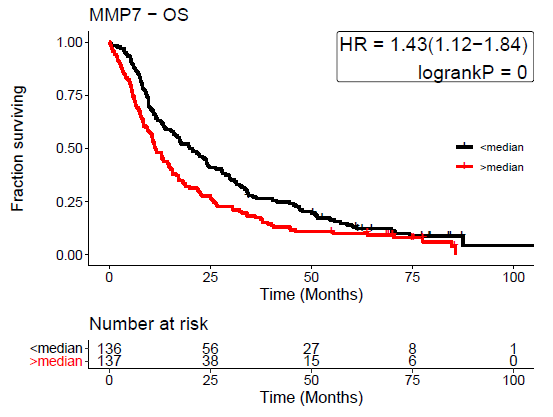


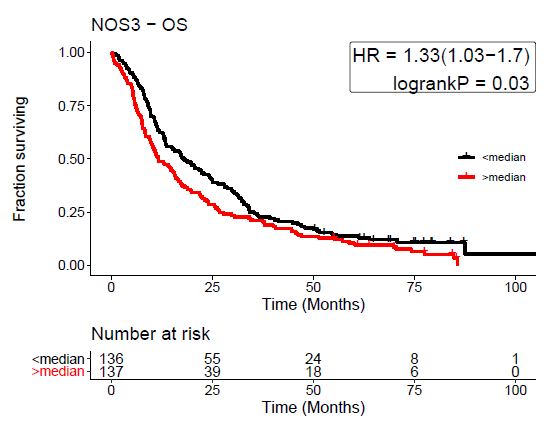

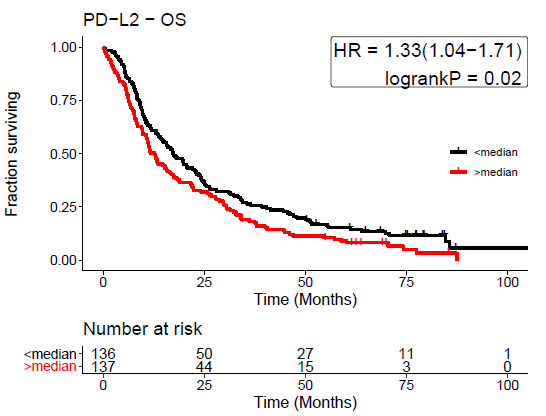


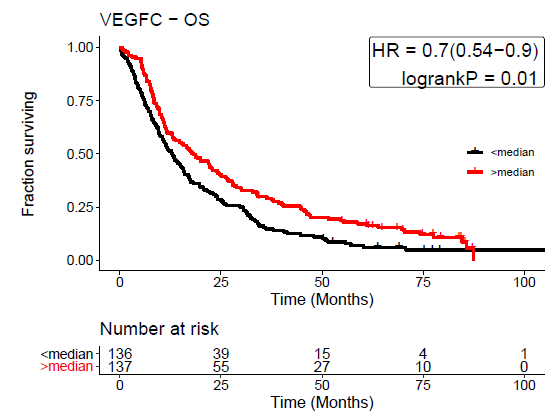


## B) Resected patients, n = 193


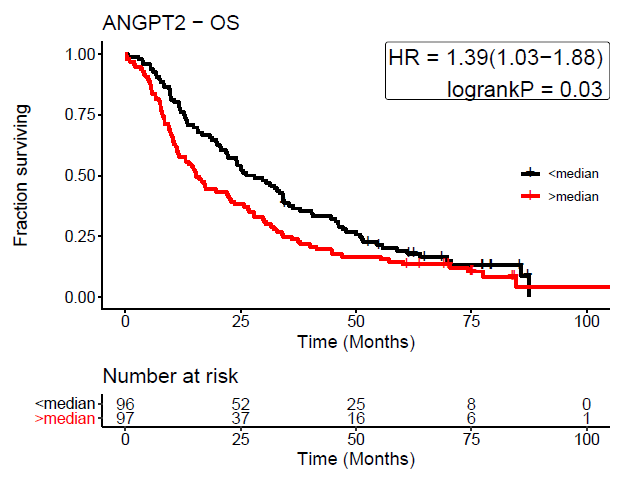

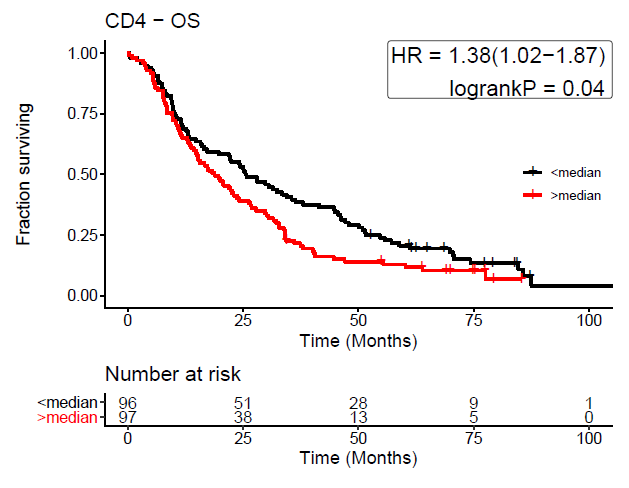


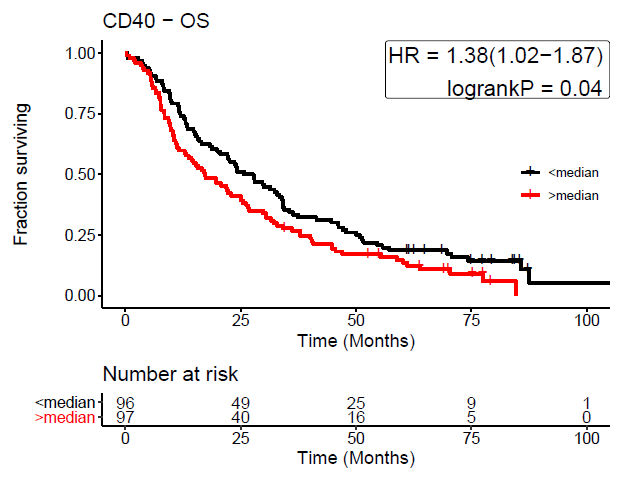

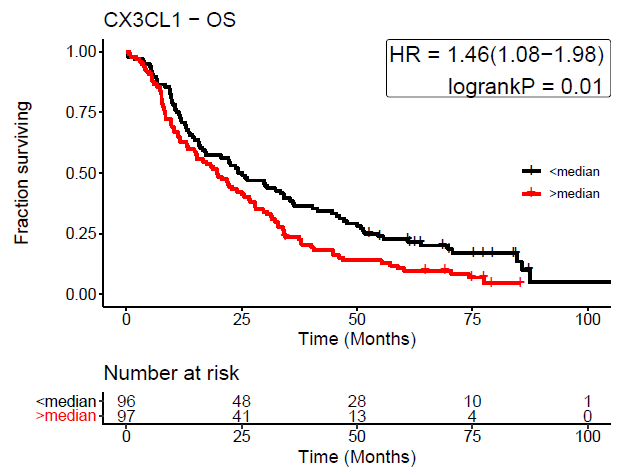


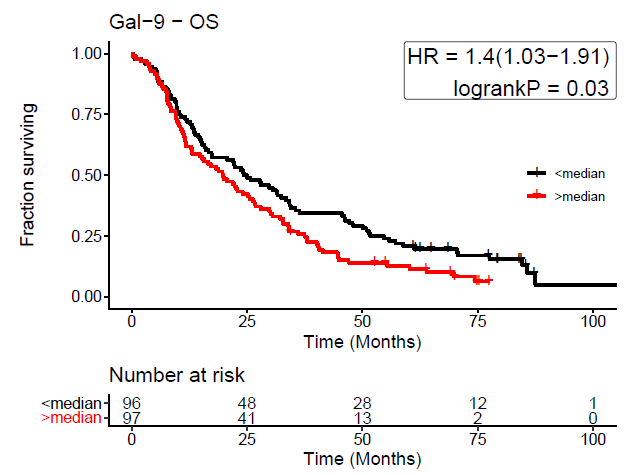

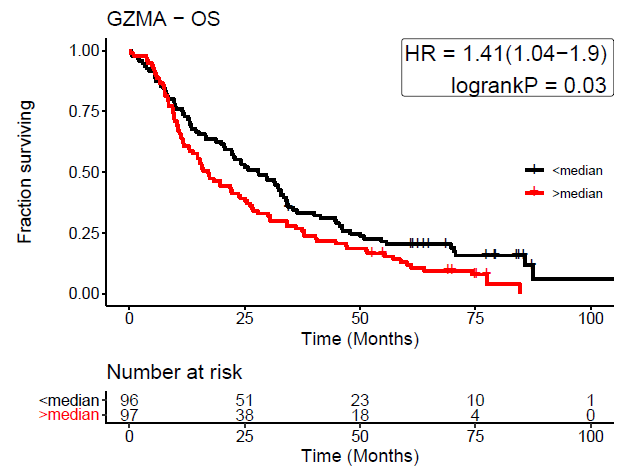


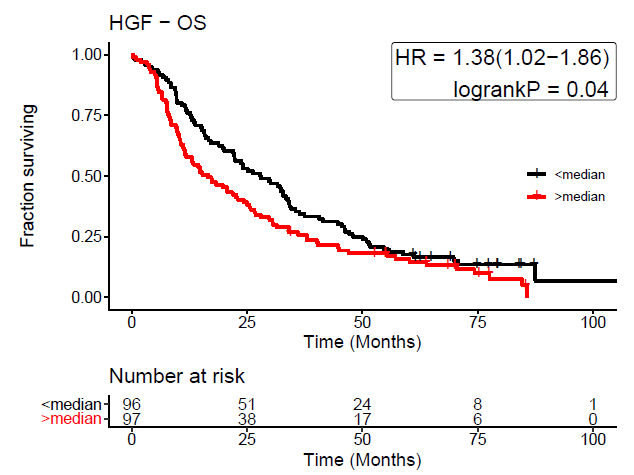

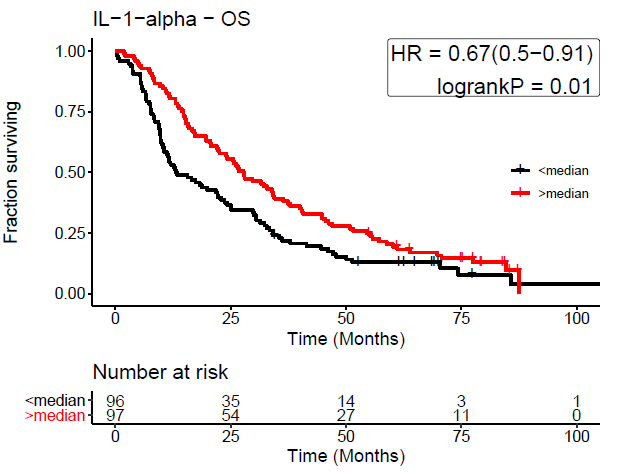


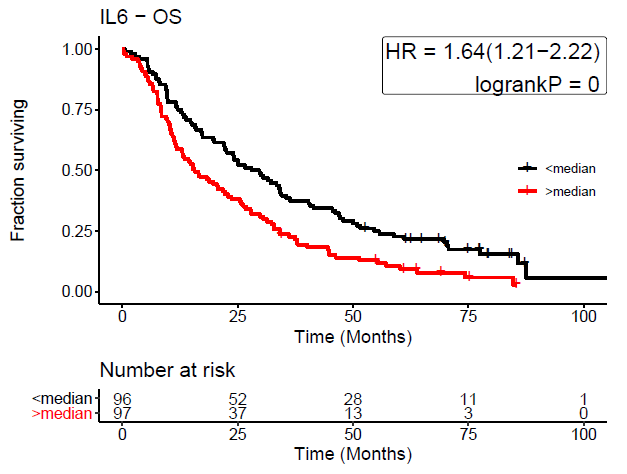

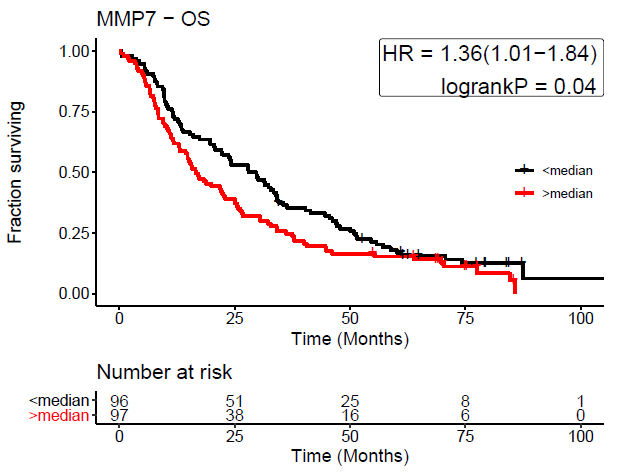


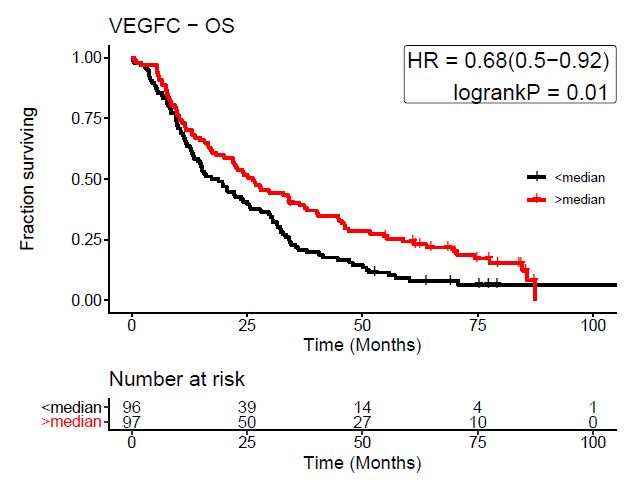


# Supplementary Figure S3: Volcano plots using unadjusted *P-*values, proteins with non-adjusted *P* < 0.05 are labeled.

a) Resectable vs. Unresectable b) Resection: OS <median vs. OS >median


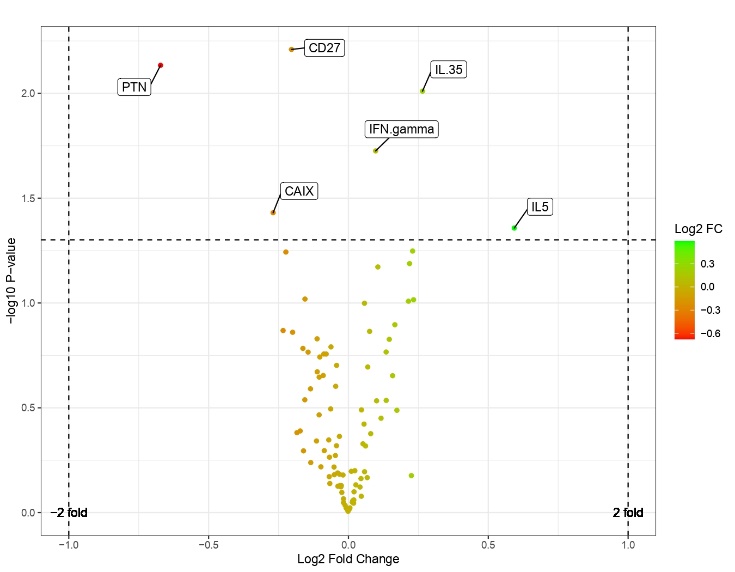

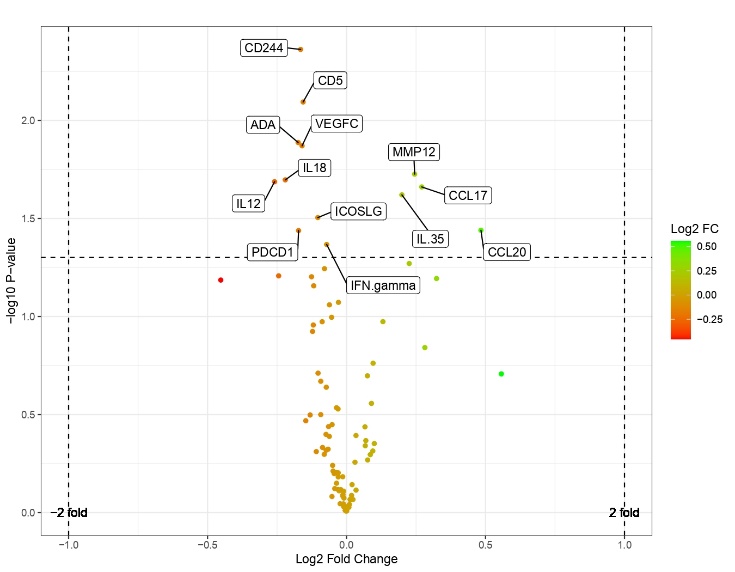


c) Unresectable: OS <median vs. OS >median d) Resection: OS <1 year vs. OS >1 year


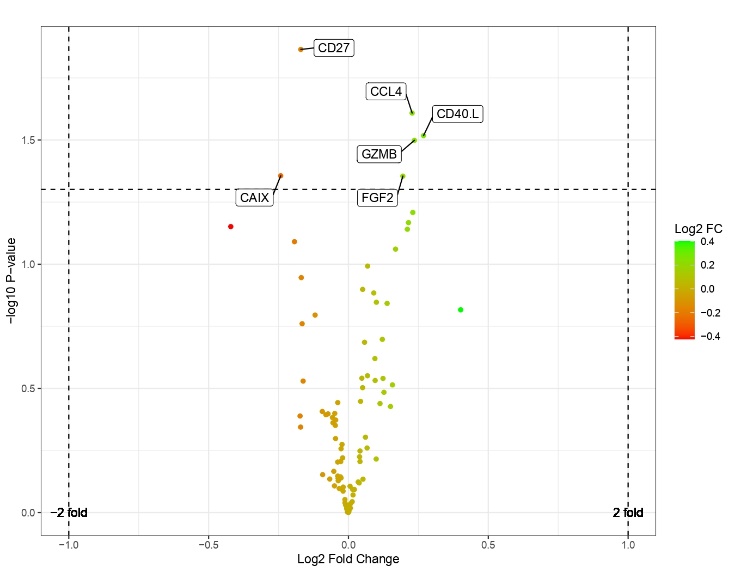

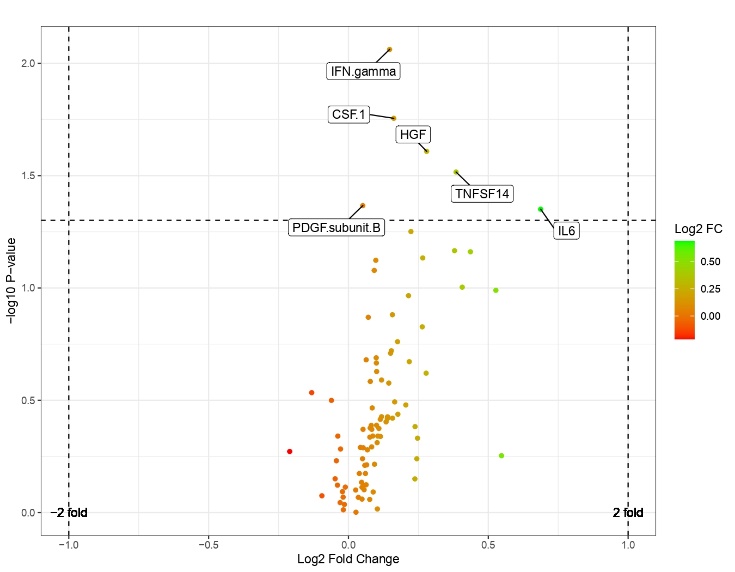


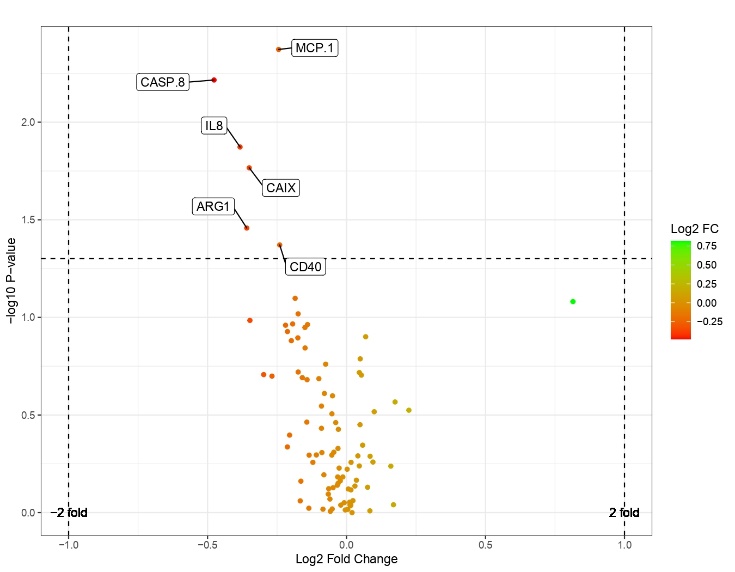
e) Resection: OS <1 year vs. OS >3 years f) Resection: OS <1 year vs. OS >4 years


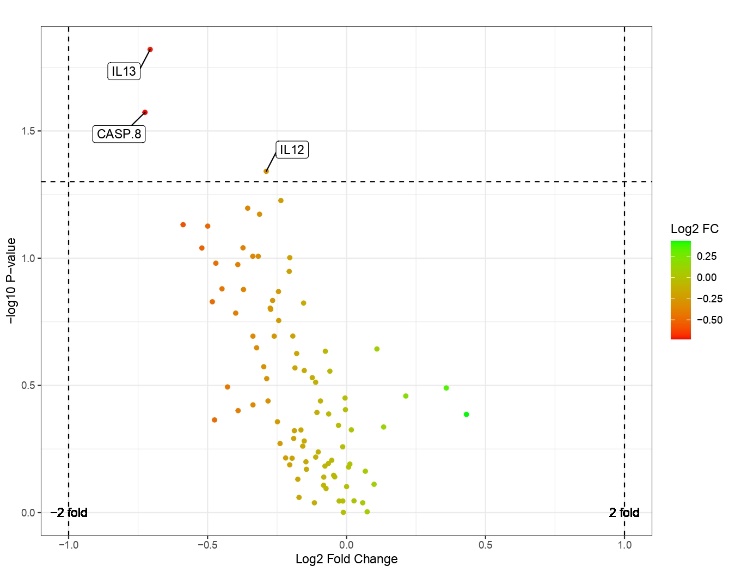


g) Resection: OS <1 year vs. OS >5 years


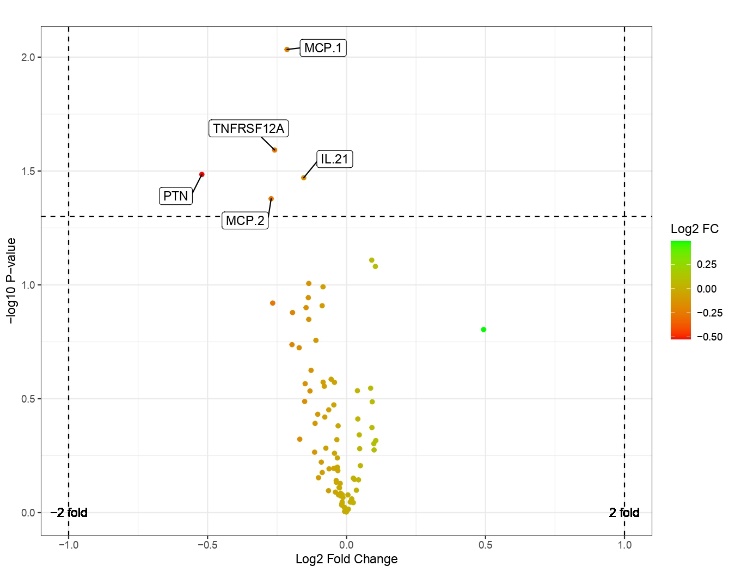


# Supplementary Figure S4: Boxplots with differential expressions of proteins with significant *P*-values (unadjusted) in the comparisons mentioned below.

**Resectable (n = 193) vs. Unresectable (n = 80):**


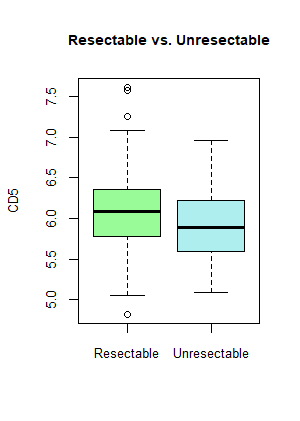
 a) ADA, *P* = 0.013 b) CCL17, *P* = 0.0218 c) CCL20, *P* = 0.0363 d) CD5, *P* = 0.00805


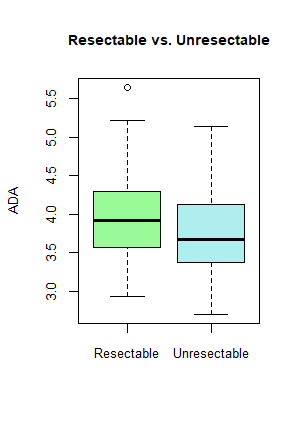

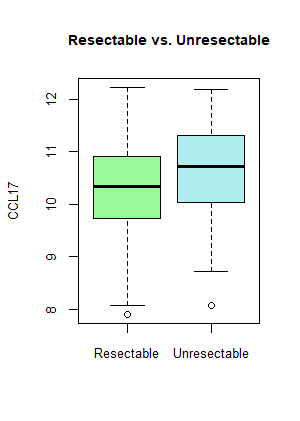

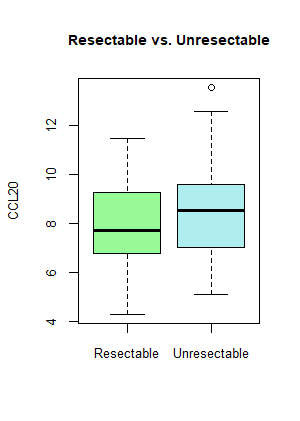


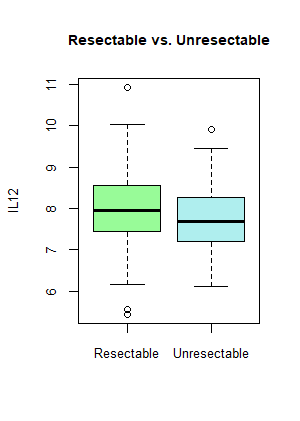
 e) CD244, *P* = 0.00435 f) ICOSLG, *P* = 0.0313 g) IFN–gamma, *P* = 0.0429 h) IL–12, *P* = 0.0205


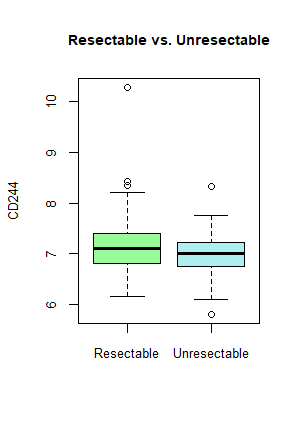

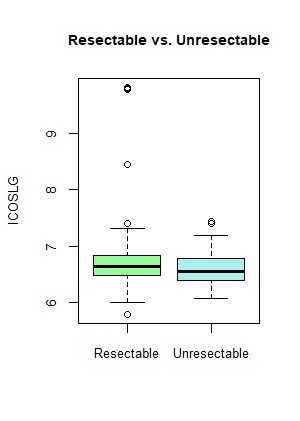

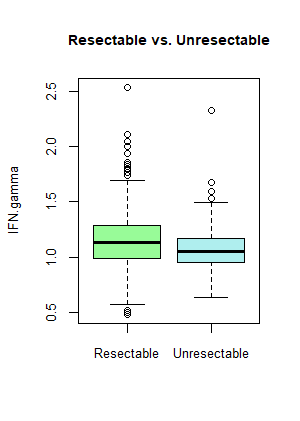


i) IL–18, *P* = 0.0201 j) IL–35, *P* = 0.024 k) MMP 12, *P* = 0.0188 l) PDCD1, *P =* 0.0365


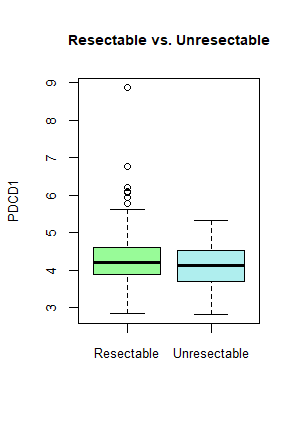

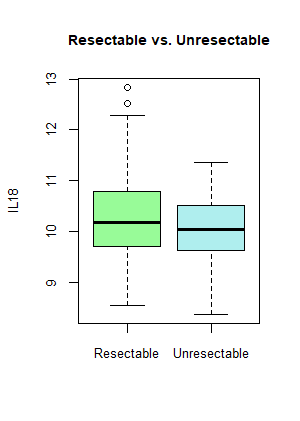

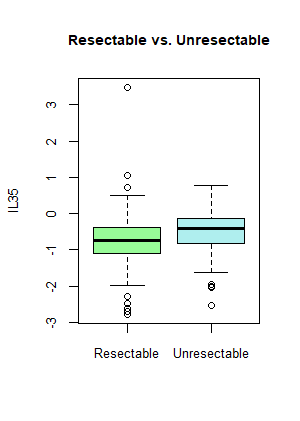

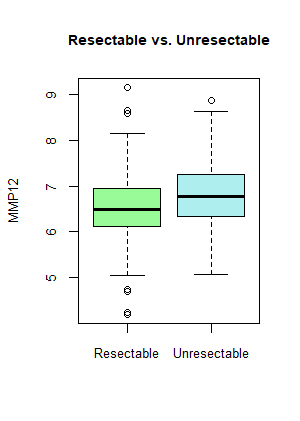


m) VEGFC, *P* = 0.0135


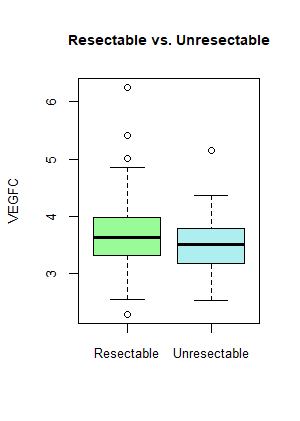


**Resectable, OS < median (n = 97) vs. > median (n = 96):**


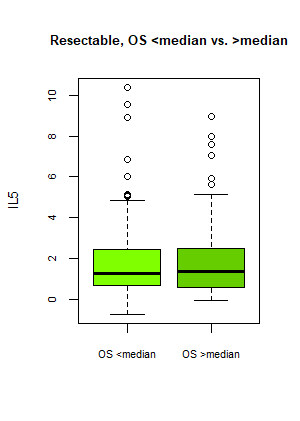
 a) CAIX, *P =* 0.0371 b) CD27, *P =* 0.00617 c) IFN–gamma, *P =* 0.0188 d) IL–5, *P =* 0.0439


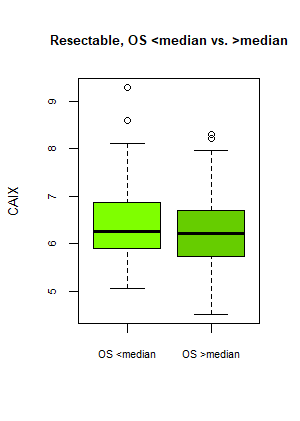

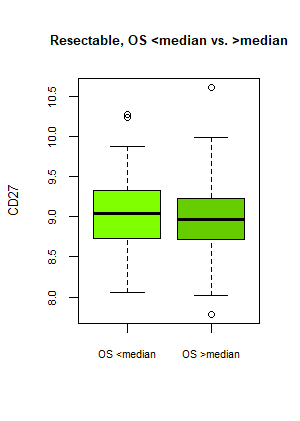

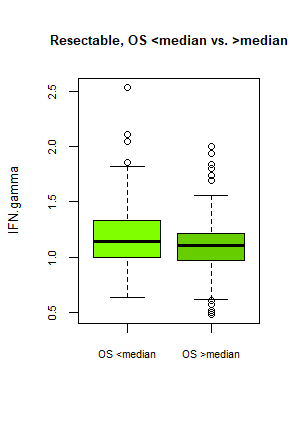


e) IL–35, *P =* 0.00976 f) PTN, *P =* 0.00735


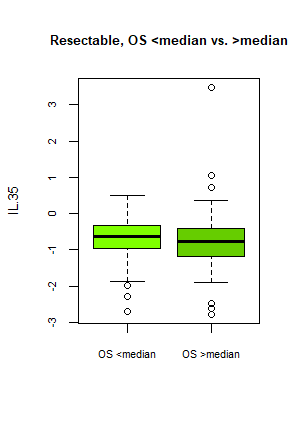

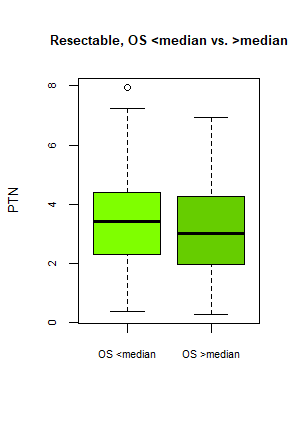


**Unresectable, OS < median (n = 40) vs. > median (n = 40):**


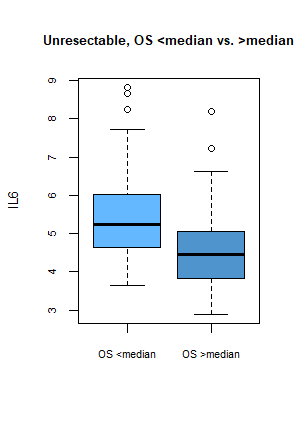
 a) CSF–1, *P =* 0.0176 b) HGF, *P =* 0.0247 c) IFN–gamma, *P =* 0.00868 d) IL–6, *P =* 0.0446


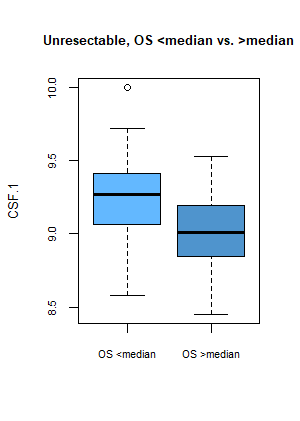

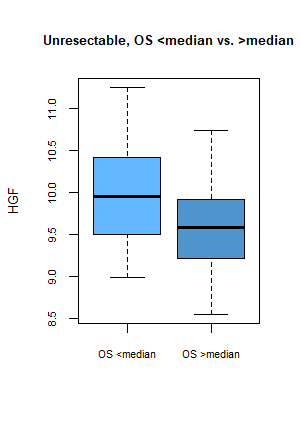

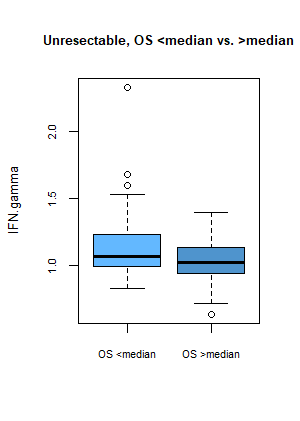


e) PDGF subunit B, *P =* 0.0430 f) TNFSF14, *P =* 0.0305


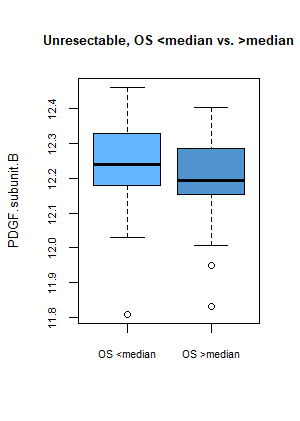

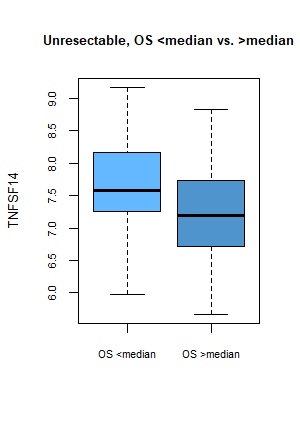


**Resectable, OS < 1 year (n = 64) vs. > 1 year (n = 129):**


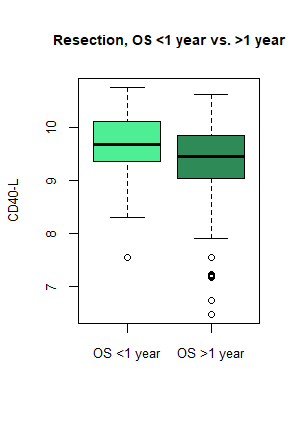
 a) CAIX, *P* = 0.0441 b) CCL4, *P* = 0.0246 c) CD27, *P* = 0.0137 d) CD40–L, *P* = 0.0304


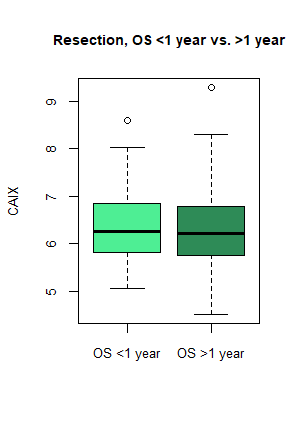

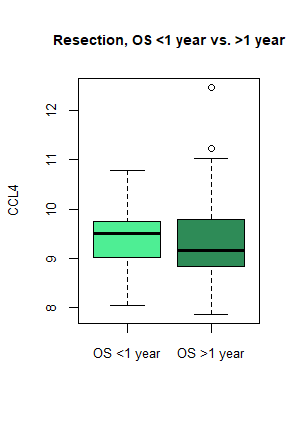

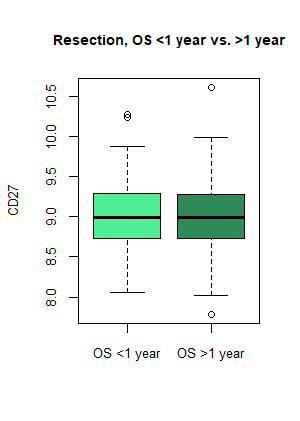


e) FGF2, *P* = 0.0442 f) GZMB, *P* = 0.0317


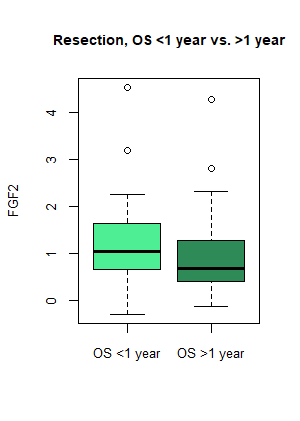

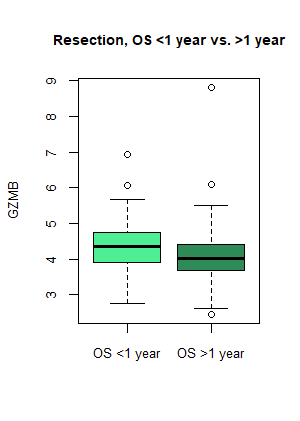


**Resectable, OS < 1 year (n = 64) vs. > 3 years (n = 59):**

a) CASP–8, *P =* 0.0267 b) IL–12, *P =* 0.0456 c) IL–13, *P =* 0.0151


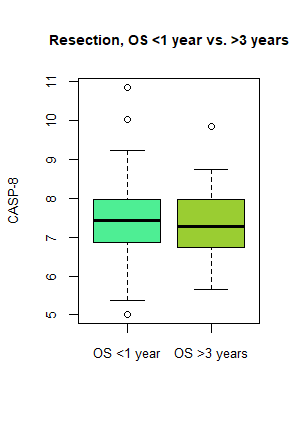

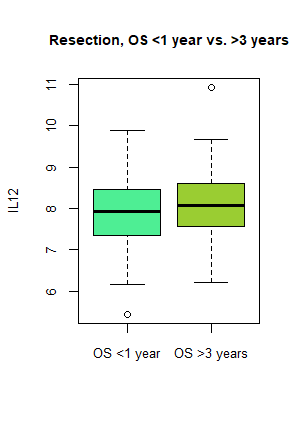

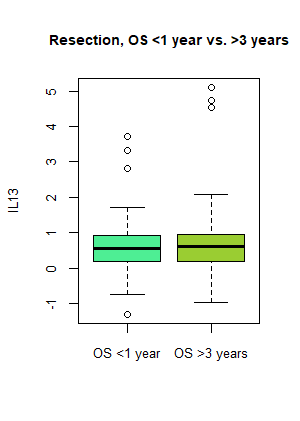


**Resectable, OS <1 year (n = 64) vs. >4 years (n = 41):**


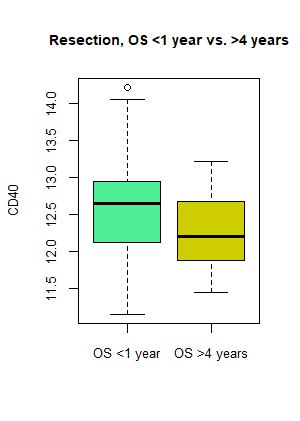
 a) ARG1, *P =* 0.0348 b) CAIX, *P =* 0.0171 c) CASP–8, *P =* 0.00608 d) CD40, *P =* 0.0426


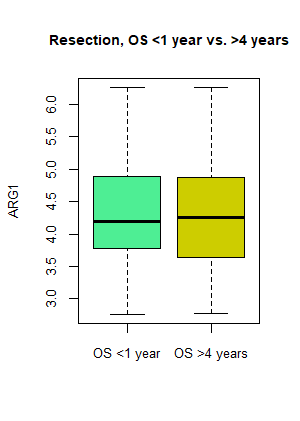

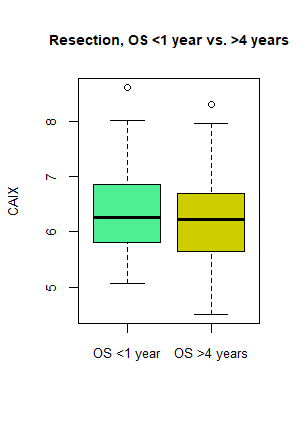

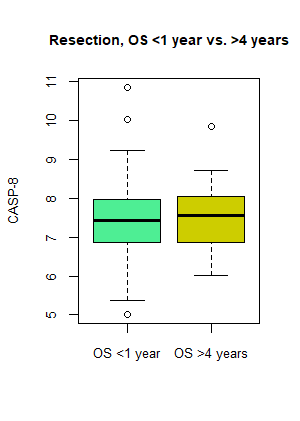


e) IL–8, *P =* 0.0134 f) MCP–1, *P =* 0.00423


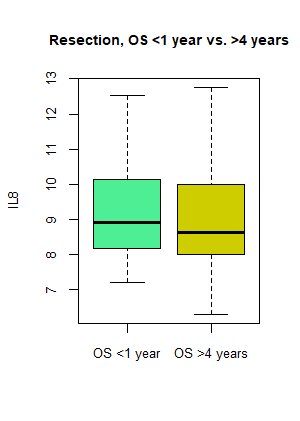

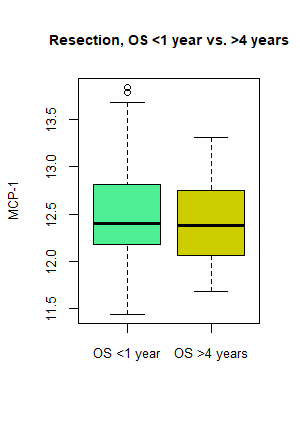


**Resectable, OS < 1 year (n = 64) vs. > 5 years (n = 30):**


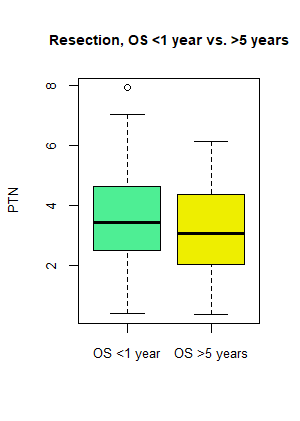
 a) IL–21, *P =* 0.0339 b) MCP–1, *P =* 0.00925 c) MCP–2, *P =* 0.0418 d) PTN, *P =* 0.0327


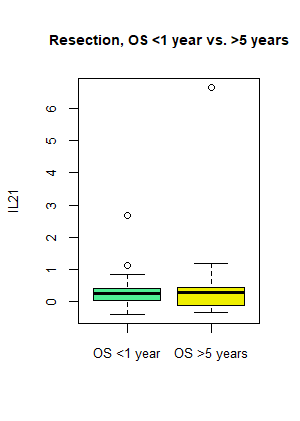

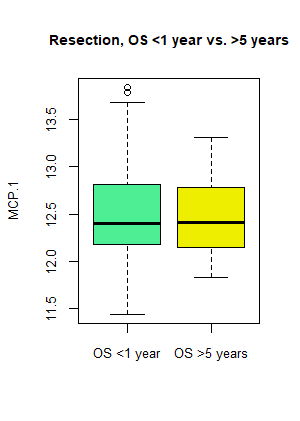

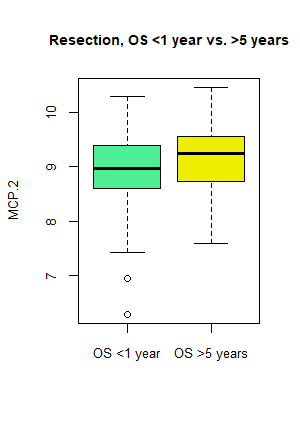


e) TNFRSF12A, *P =* 0.0256


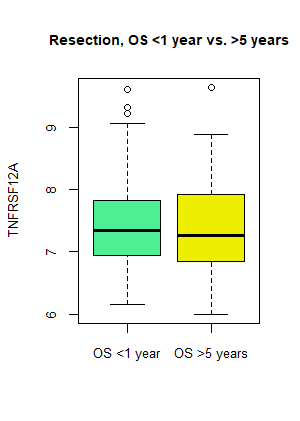


# Supplementary Figure S5: Principal component analysis dividing patients according to resection.


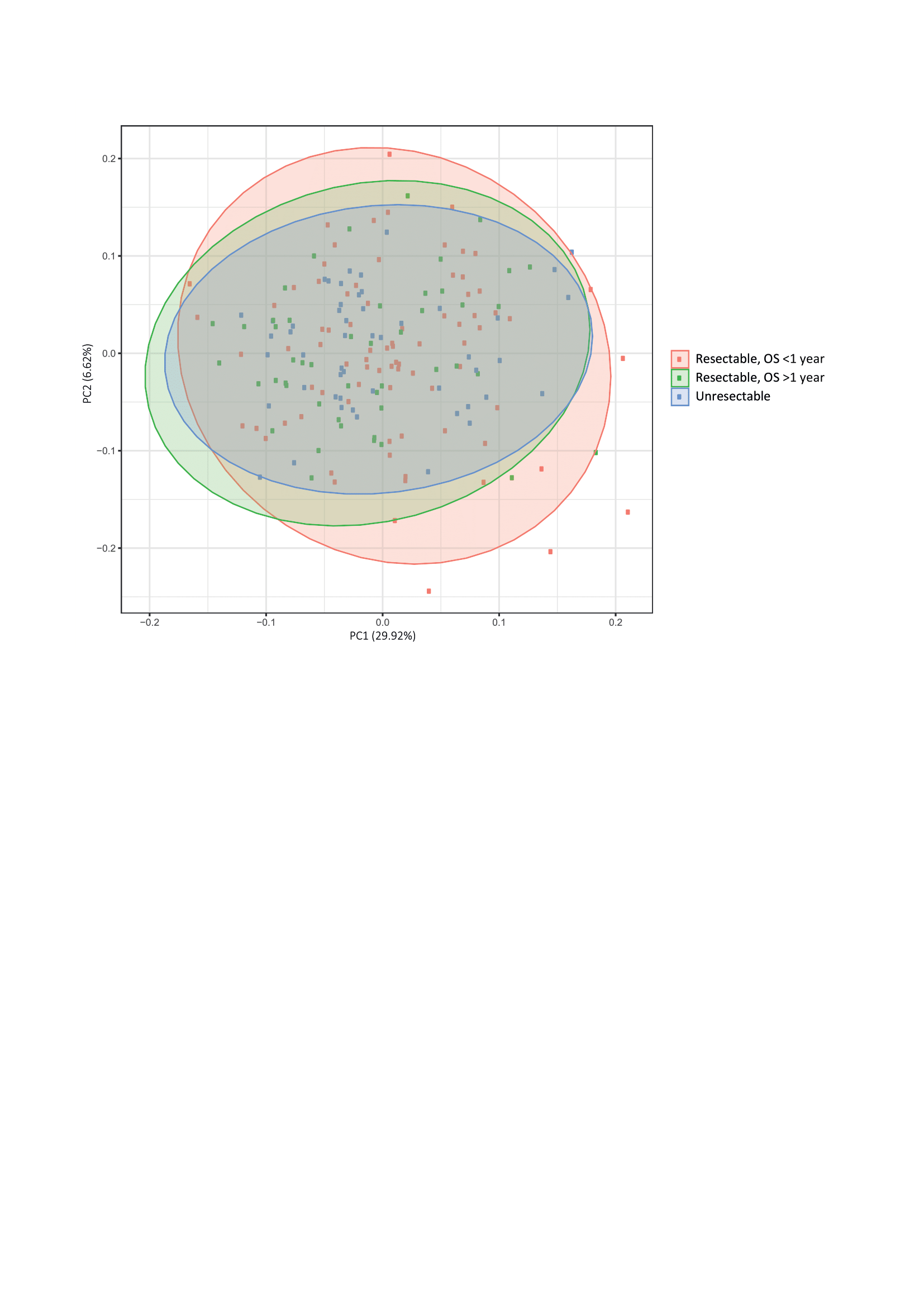


# Supplementary Figure S6: ROC plot for differentiation between resectable and unresectable patients.

a) ROC plot


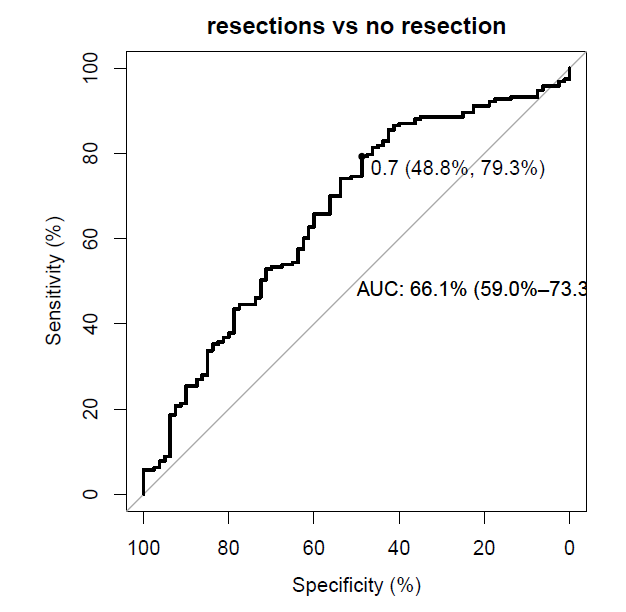


b) Weights for the proteins included in the signature

| **Predictor** | **Weight** |
| --- | --- |
| IL–12 | 0.0366291 |
| MIC–A/B | 0.0395936 |
| ADA | 0.0472037 |
| CD244 | 0.0570681 |
| CD5 | 0.0751803 |
| VEGFC | 0.0950826 |
| intercept | 0.8895062 |

# Supplementary Figure S7: Prediction score vs. survival for Index IV.

a) Index IV, prediction score vs. survival


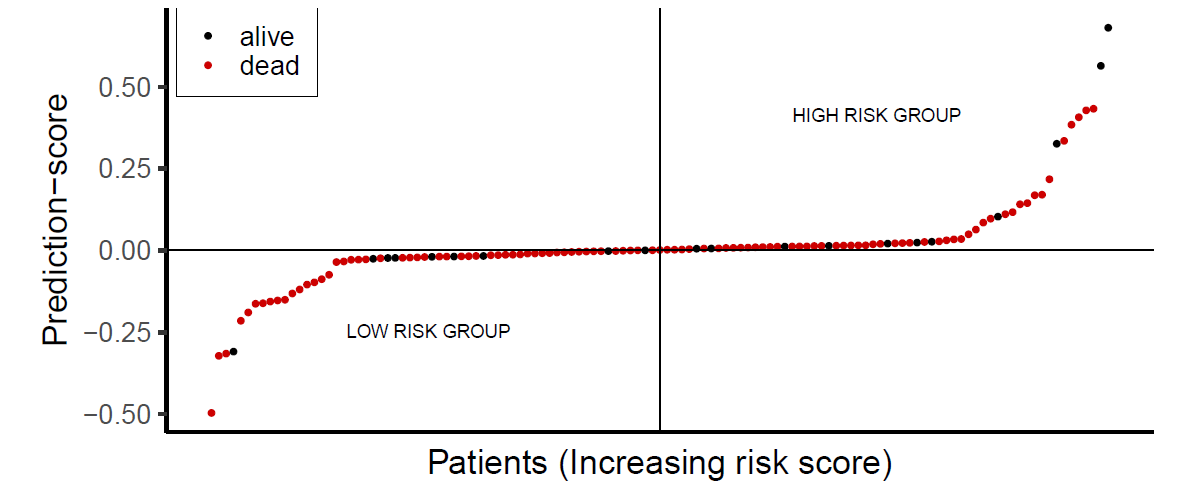


b) Index IV, prediction score vs. survival, Kaplan–Meier plot, median risk score cutoff


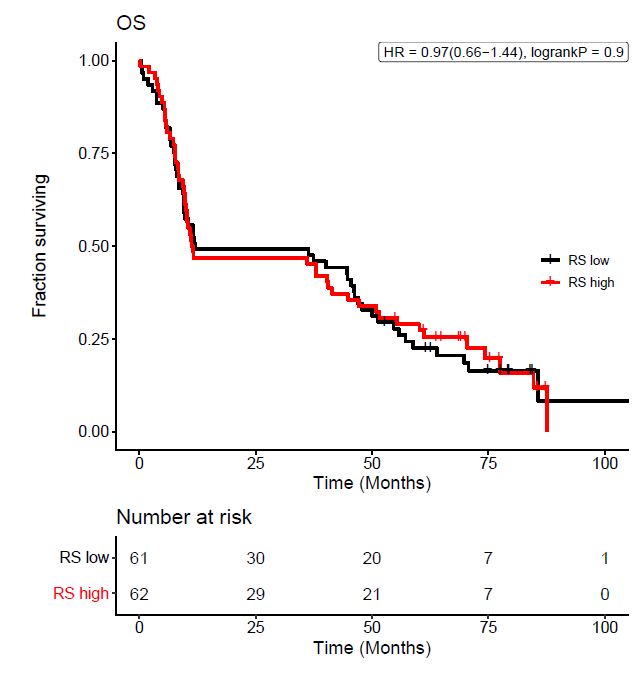


c) Index IV, prediction score vs. survival, Kaplan–Meier plot, best point risk score cutoff


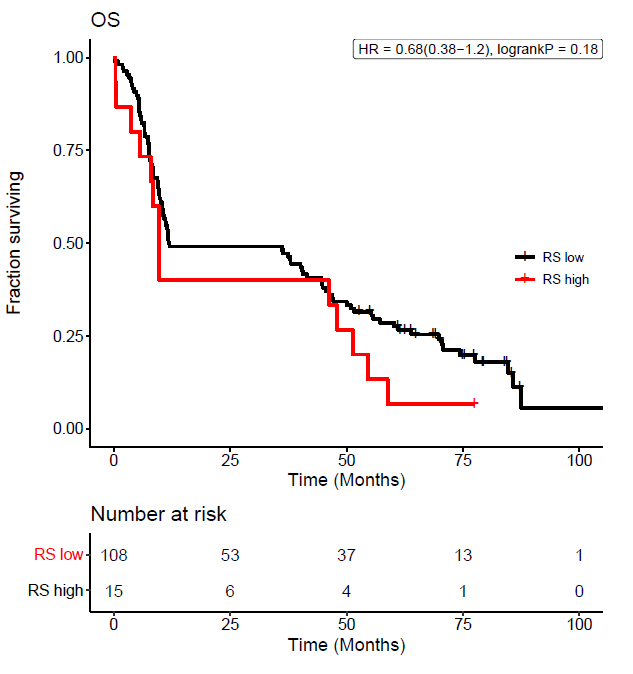


# References

1. Enroth S, Berggrund M, Lycke M, Broberg J, Lundberg M, Assarsson E, Olovsson M, Stålberg K, Sundfeldt K, Gyllensten U. High throughput proteomics identifies a high–accuracy 11 plasma protein biomarker signature for ovarian cancer. Commun Biol 2019;2:221–21.

2. Lindgaard SC, Sztupinszki Z, Maag E, Chen IM, Johansen AZ, Jensen BV, Bojesen SE, Nielsen DL, Hansen CP, Hasselby JP, Nielsen KR, Szallasi Z, et al. Circulating Protein Biomarkers for Use in Pancreatic Ductal Adenocarcinoma Identification. Clinical cancer research : an official journal of the American Association for Cancer Research 2021;27:2592–603.

3. McShane LM, Altman DG, Sauerbrei W, Taube SE, Gion M, Clark GM. REporting recommendations for tumour MARKer prognostic studies (REMARK). Br J Cancer 2005;93:387–91.
